# Challenging immunodominance of influenza-specific CD8^+^ T cell responses restricted by the risk-associated HLA-A*68:01 allomorph

**DOI:** 10.1038/s41467-019-13346-4

**Published:** 2019-12-06

**Authors:** C. E. van de Sandt, E. B. Clemens, E. J. Grant, L. C. Rowntree, S. Sant, H. Halim, J. Crowe, A. C. Cheng, T. C. Kotsimbos, M. Richards, A. Miller, S. Y. C. Tong, J. Rossjohn, T. H. O. Nguyen, S. Gras, W. Chen, K. Kedzierska

**Affiliations:** 1 Department of Microbiology and Immunology, University of Melbourne at The Peter Doherty Institute, Melbourne, VIC 3000 Australia; 2 Department of Hematopoiesis, Sanquin Research and Landsteiner Laboratory, Amsterdam UMC, University of Amsterdam, 1066CX Amsterdam, Netherlands; 3 Infection and Immunity Program and The Department of Biochemistry and Molecular Biology, Biomedicine Discovery Institute, Monash University, Clayton, VIC 3800 Australia; 4 Deepdene Surgery, Deepdene, VIC 3103 Australia; 5 School of Public Health and Preventive Medicine, Monash University, Melbourne, VIC 3004 Australia; 6 Infection Prevention and Healthcare Epidemiology Unit, Alfred Health, Melbourne, VIC 3004 Australia; 7 Department of Allergy, Immunology and Respiratory Medicine, The Alfred Hospital, Melbourne, VIC 3004 Australia; 8 Department of Medicine, Monash University, Central Clinical School, The Alfred Hospital, Melbourne, VIC 3004 Australia; 9 Victorian Infectious Diseases Service, The Royal Melbourne Hospital, at the Peter Doherty Institute for Infection and Immunity, Parkville, VIC 3050 Australia; 10 Indigenous Research Network, Griffith University, Brisbane, QLD 4222 Australia; 11 Menzies School of Health Research, Charles Darwin University, Darwin, NT 0811 Australia; 12 Australian Research Council Centre of Excellence for Advanced Molecular Imaging, Monash University, Clayton, VIC Australia; 13 Institute of Infection and Immunity, Cardiff University School of Medicine, Heath Park, Cardiff, CF14 4XN United Kingdom; 14 Department of Biochemistry and Genetics, La Trobe Institute of Molecular Science, La Trobe University, Bundoora, VIC 3086 Australia; 15 Present Address: Department of Biochemistry and Molecular Biology, Biomedicine Discovery Institute, Infection and Immunity Program, Monash University, Clayton, VIC 3800 Australia; 16 Present Address: Office of Indigenous Engagement, CQUniversity, Townsvillle, QLD Australia

**Keywords:** Antigen presentation, Antimicrobial responses, Influenza virus, CD8-positive T cells

## Abstract

Although influenza viruses lead to severe illness in high-risk populations, host genetic factors associated with severe disease are largely unknown. As the HLA-A*68:01 allele can be linked to severe pandemic 2009-H1N1 disease, we investigate a potential impairment of HLA-A*68:01-restricted CD8^+^ T cells to mount robust responses. We elucidate the HLA-A*68:01^+^CD8^+^ T cell response directed toward an extended influenza-derived nucleoprotein (NP) peptide and show that only ~35% individuals have immunodominant A68/NP_145_^+^CD8^+^ T cell responses. Dissecting A68/NP_145_^+^CD8^+^ T cells in low vs. medium/high responders reveals that high responding donors have A68/NP_145_^+^CD8^+^ memory T cells with clonally expanded TCRαβs, while low-responders display A68/NP_145_^+^CD8^+^ T cells with predominantly naïve phenotypes and non-expanded TCRαβs. Single-cell index sorting and TCRαβ analyses link expansion of A68/NP_145_^+^CD8^+^ T cells to their memory potential. Our study demonstrates the immunodominance potential of influenza-specific CD8^+^ T cells presented by a risk HLA-A*68:01 molecule and advocates for priming CD8^+^ T cell compartments in HLA-A*68:01-expressing individuals for establishment of pre-existing protective memory T cell pools.

## Abstract (web summary)

The HLA-A*68:01 allele has been associated with severe influenza disease during the 2009 influenza pandemic. Here, the authors analyze influenza nucleoprotein specific HLA-A*68:01-restricted CD8^+^ T cells from human donors and show immunodominance of these cells in approximately 35% of HLA-A*68:01-expressing donors.

## Introduction

Although 2018 marked the 100th anniversary of the Spanish influenza pandemic, which killed >50 million people^1^, influenza viruses remain a constant global health threat. Indeed, a global influenza pandemic is listed as one of the WHO Top Ten Global Health Threats in 2019^2^. The next influenza pandemic outbreak is inevitable and the mechanisms leading to differential disease outcomes are unclear. Therefore, it is important to understand why some individuals succumb to severe and fatal influenza disease during pandemic outbreaks and seasonal epidemics^1,3^.

Although current antibody-based vaccines targeting variable hemagglutinin (HA) and neuraminidase (NA) surface glycoproteins are the most effective way to combat seasonal infections, they fail during an influenza pandemic caused by the emergence of an antigenically distinct influenza virus subtype^4,5^. In the absence of protective antibodies, a novel influenza A virus (IAV) can activate and recall memory cross-strain protective cytotoxic CD8^+^ T cells, specific for conserved viral peptides^6–11^, resulting in rapid viral clearance and reduced disease severity^12,13^. This makes cross-reactive CD8^+^ T cells an attractive target for novel universal influenza vaccine strategies^5^. Following the 2013 H7N9 outbreak in China, we illustrated the importance of robust pre-existing cytotoxic CD8^+^ T cells memory for protection against severe influenza disease (and death) caused by novel IAVs^13,14^. Our recent study introduces a new paradigm, whereby human CD8^+^ T cells confer unprecedented cross-reactivity across all influenza A, B, and C viruses, having key implications for the design of universal vaccines that do not require annual reformulation^11^. Vaccines eliciting cross-reactive CD8^+^ T cells would reduce annual rates of influenza A and B virus-induced morbidity/mortality, protect children from influenza C virus, and augment CD8^+^ T cells in people with previous influenza exposures^5,11^.

CD8^+^ T cells recognize virus-derived peptides in the context of human leukocyte antigen (HLA) class I molecules via their T cell receptors (TCRs) to specifically eliminate virus-infected cells^15^. HLA-A*01:01, HLA-A*02:01, HLA-A*03:01, HLA-B*08:01, HLA-B*18:01, HLA-B*27:05, HLA-B*37:01, and HLA-B*57:01^9–11^ are associated with universal protective CD8^+^ T cell mediated immunity, as they present influenza peptides that were highly conserved over the last century. Conversely, two independent studies associated the HLA-A*68:01 allele, which is expressed at 5.2–25% allele frequency, with severe influenza disease during the 2009 influenza pandemic^16,17^. To understand CD8^+^ T cell responses in the context of the high risk HLA-A*68:01 molecule, we have recently identified a novel extended 12 amino acid influenza-derived peptide from the virus nucleoprotein (NP_145–__156_; DATYQRTRALVR) with the capacity to bind to HLA-A*68:01^18^.

HLA class I molecules predominantly bind short viral peptides (8–10 amino acids; aa)^19^, although extended peptides (>10 aa) can be presented to CD8^+^ T cells in the context of HLA class I^19–21^. Similar to canonical length peptides (8–10 aa), longer peptides (≥11 aa) have similar anchor residues at the second (P2) and last position (PΩ). As a result, the central region of these extended peptides is forced to bulge from the HLA class I antigen-binding cleft^19,20^, although >11 aa peptides that extent from the N- or C-terminus have been described^22,23^. The bulging conformation of the extended HLA class I-restricted peptides may prove especially challenging for TCR recognition^24,25^. Based on a limited amount of studies on long peptide-specific TCR repertoires, it appears that long peptide-HLA class I complexes drive a biased TCR gene usage, suggesting that bulging peptide-HLA class I complexes can only be recognized by few TCRs, which may affect CD8^+^ T cell recruitment^26–31^. Conversely, the bulging part of the peptide can also display high mobility, providing multiple TCR binding sites, thus selecting a diverse TCR repertoire^32,33^. Indeed, a diverse TCR repertoire has been demonstrated for two long peptides (HLA-B*07:02/NY-ESO-1 (13 aa) and HLA-B*57:03-KF11 (11 aa))^20,30,34^.

In the present study, we elucidate the contribution of CD8^+^ T cell responses directed toward this novel extended HLA-A*68:01-NP_145-156_ (hereafter A68/NP_145_) epitope to the overall influenza-specific immunity. Understanding the role of CD8^+^ T cells presenting influenza viral peptides in the context of risk HLA-I molecules such as HLA-A*68:01 is of key importance to rationally design universal T cell-targeted influenza virus vaccines. Here, we provide an in-depth analysis of influenza virus-specific CD8^+^ T cells directed against the extended 12 aa NP_145_ peptide restricted by the risk HLA-A*68:01 molecule. Our data show an immunodominance potential of influenza-specific CD8^+^ T cells in the context of a risk HLA-A*68:01 molecule in 35% donors and advocates for priming CD8^+^ T cell compartments in HLA-A*68:01-expressing individuals for establishment of pre-existing protective memory CD8^+^ T cell pools against future unpredicted influenza strains.

## Results

### Structural flexibility of NP_145_ peptide bound to HLA-A*68:01

**Fig. 1:**
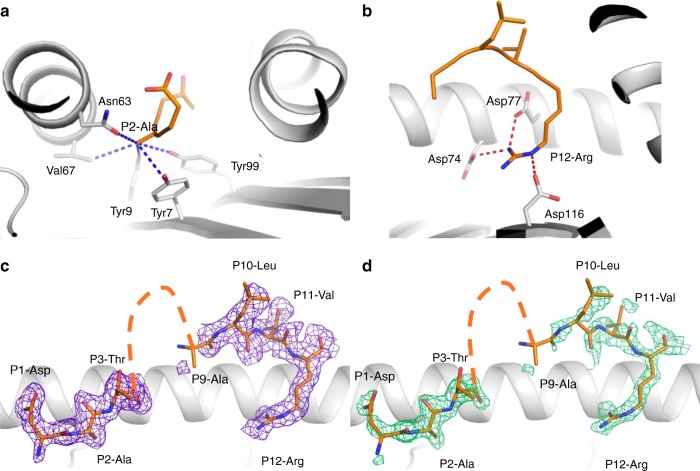
Structure of the HLA-A*68:01-NP_145_ complex. Structure of the HLA-A*68:01 (white cartoon) bound to the NP_145_ peptide (orange stick). **a** A zoomed view of the P2-Ala anchor residue interaction with the HLA-A*68:01 molecule, the blue dashed lines represent hydrophobic interactions. **b** The P12-Arg salt bridge network (red dashed lines) with the HLA-A*68:01 amino acids. **c**, **d** The electron density maps after refinement (**c** 2Fo-Fc map colored in purple and contoured at 1 σ) or before building the peptide in **d** (Fo-Fc or omit map colored in green and contoured at 3 σ). The orange dashed line on **c**, **d** represent the missing amino acid from the NP_145_ peptide (P4–P8) in the crystal structure.

An influenza-derived 12 aa peptide NP_145_ (DATYQRTRALVR), presented by an influenza risk HLA-I allomorph HLA-A*68:01^18^, is one of a relatively few immunogenic (derived from pathogens) human CD8^+^ T cell peptides over 11 aa reported to date^19^. To understand whether presentation of this extended NP_145_ peptide (hereafter NP_145_) within HLA-A*68:01 was associated with any structural constraints, which potentially could affect TCR recognition of the A68/NP_145_ epitope, we solved the structure of the HLA-A*68:01-NP_145_ complex at a resolution of 1.90 Å (Supplementary Table 1). The structure showed that the anchor residue Ala at P2 was bound within the HLA-A*68:01 peptide-binding groove, despite being smaller than the usual P2 anchor residues for HLA-A*68:01 (Val or Thr) (Fig. 1a). The C-terminal part of the NP_145_ peptide possessed a canonical HLA-A*68:01 anchor residue Arg at P12, which formed a network of salt bridges with Asp77, Asp74, and Asp114 within the HLA-A*68:01 molecule (Fig. 1b). In contrast, the central section (residues P4–P9) of the NP_145_ peptide presented weak/no electron density associated with a high degree of peptide mobility, despite the well-defined electron density of the N- and C-terminal ends of the NP_145_ peptide (Fig. 1c, d). The flexible nature of the NP_145_ peptide might potentially present a challenge for TCR engagement, thereby limiting the recruitment of A68/NP_145_-specific CD8^+^ T cells during influenza virus infection.

### Conservation of the NP_145_ peptide across IAVs

**Fig. 2:**
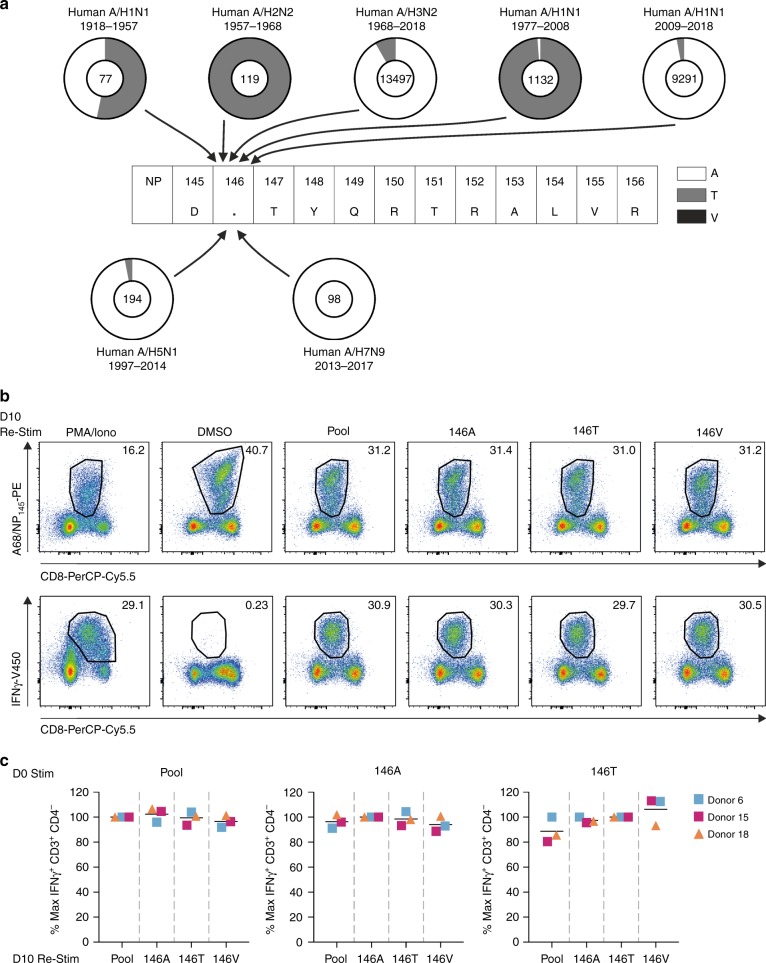
High conservation of the NP_145-156_ peptide across seasonal IAV viruses. **a** Frequency of amino acid variation at position 146 of the NP_145–156_ peptide. Pie charts represent frequency of alanine (A; white), threonine (T; gray) and valine (B, black) at position 146. Total number of analyzed sequences are indicated in the pie charts: A/H1N1 (1918–1957 *n* = 77, 1977–2008 *n* = 1132, 2009–2018 *n* = 9291), A/H2N2 (1957–1968 *n* = 119), and A/H3N2 (1986–2018 *n* = 13,497), avian A/H5N1 (1997–2014 *n* = 194) and A/H7N9 (2013–2017 *n* = 98). **b** Representative FACS panels of A68/NP_145_-specific CD8^+^ T cells expanded by 146A peptide stimulation on day 0, followed by restimulation at day 10 with cognate or variant NP_145_ peptides. Day 10 restimulation with PMA/Ionomycin was included as a positive control and DMSO as a negative control. **c** Percentage of maximum IFNγ^+^CD3^+^CD4^−^ T cells following secondary stimulation. Maximum IFNγ is defined as IFNγ^+^CD3^+^CD4^−^ T cells following cognate restimulation with peptide pool (pool), D**A**TYQRTRALVR (146A), D**T**TYQRTRALVR (146T) or D**V**TYQRTRALVR (146V) peptides, respectively. Pool included 146A, 146T, and 146V variants. The bar indicates the mean response of three donors (*n* = 3).

As variations within the viral peptide can affect peptide-HLA class I binding and presentation^15,35–39^, as well as TCR recognition^9,15,40,41^, we next assessed the conservation level of the NP_145_ peptide within the influenza viruses. We examined 24,408 nucleoprotein (NP) sequences derived from human A/H1N1 (1918–1957 *n* = 77, 1977–2008 *n* = 1132, 2009–2018 *n* = 9291), A/H2N2 (1957–1968 *n* = 119) and A/H3N2 (1986–2018 *n* = 13,497) viruses as well as avian A/H5N1 (1997–2014 *n* = 194), and A/H7N9 (2013–2017 *n* = 98) viruses (human isolates) (NCBI; http:/www.ncbi.nlm.nih.gov/genomes/FLU database, accessed on July 31, 2018). Based on our sequence alignments, we found that while amino acids at positions 145, 147–156 within the viral NP_145_ peptide were highly conserved (Supplementary Table 2), variability was observed at position 146 (P2 of the peptide), with influenza viruses expressing either an alanine (A) (21,740/24,408 viruses, 89.1%) or threonine (T) (2661/24,408 viruses, 10.9%), and to a lesser extent a valine (V) (7/24,408 viruses; <0.01% (Supplementary Table 2; Fig. 2a). Chronological analyses of the residues at position 146 showed that these variations were not randomly distributed, but instead became fixed with time. While the earliest known influenza virus isolates from 1918 had an alanine at position 146, this was replaced by an A146T substitution in 1935. The 146T variant of the NP_145–146_ peptide was passed onto the A/H2N2 and subsequently the A/H3N2 viruses by two reassortment events^1^. The 146T variant of the NP_145_ peptide continued to circulate until 2001 when the T146A substitution was rapidly fixed and continues to circulate until now. The 1957 A/H1N1 virus (146T) was reintroduced in the human population in 1977 and continued to circulate up to 2009, when it was replaced by a multiple reassorted A/H1N1 virus that contained the original NP gene segment from 1918 (146A)^1^ (Fig. 2a). With the exception of the A/Shandong/1/2009 strain, all human viral isolates of the avian A/H5N1 and A/H7N9 viruses express an alanine at position 146 (Fig. 2a, Supplementary Table 2).

Despite virus antigenic variations, influenza-specific CD8^+^ T cells can provide broad cross-reactivity and recognize an array of peptide variants^6,8,10,15,41–44^. Thus, we analysed the cross-reactive potential of A68/NP_145_^+^CD8^+^ T cells toward the two main NP_145_ variants, D**A**TYQRTRALVR and D**T**TYQRTRALVR, as well as the less prevalent D**V**TYQRTRALVR variant. Given that the 146A and 146T variants of the NP_145-_ peptide have predominantly circulated in the last two decades, both NP_145_ peptide variants were able to expand A68/NP_145_^+^CD8^+^ T cells (Fig. 2b, c, Supplementary Fig. 1a). The FACS plots are representatives of 146A peptide-stimulated and expanded A68/NP_145_-specific CD8^+^ T cells at day 10. DMSO was used as a negative control for the second 6-h restimulation in an IFN-γ ICS assay of these expanded cells, thus the large population of tetramer-positive cells resulted from the initial expansion after the first stimulation (Fig. 2b). However, negligible IFN-γ production was detected in the DMSO control (Fig. 2b). Re-stimulating the expanded A68/NP_145_^+^CD8^+^ T cells with three variants of the NP_145_ peptide (146A, 146T, and 146V) revealed a substantial level of cross-reactivity toward all three peptides (Fig. 2c, *n* = 3 donors). The high level of cross-reactivity between the 146A, 146T, and 146V peptide variants suggests that fixation of these mutations at position 146 did not result in viral escape from pre-existing A68/NP_145_^+^CD8^+^ T cell responses and would therefore not be a determining factor in HLA^−^A*68:01-associated morbidity when a new variant is introduced.

Since the NP_145_-146A variant of the NP_145_ peptide circulated in the past two decades, and is cross-reactive with the other variants, we selected this immunogenic DATYQRTRALVR peptide to further dissect the quantitative, qualitative, and clonal characteristics of A68/NP_145_-specific CD8^+^ T cell responses in HLA-A*68:01-expressing individuals.

### A68/NP_145_-^+^CD8^+^ T cell responses vary across the donors

**Fig. 3:**
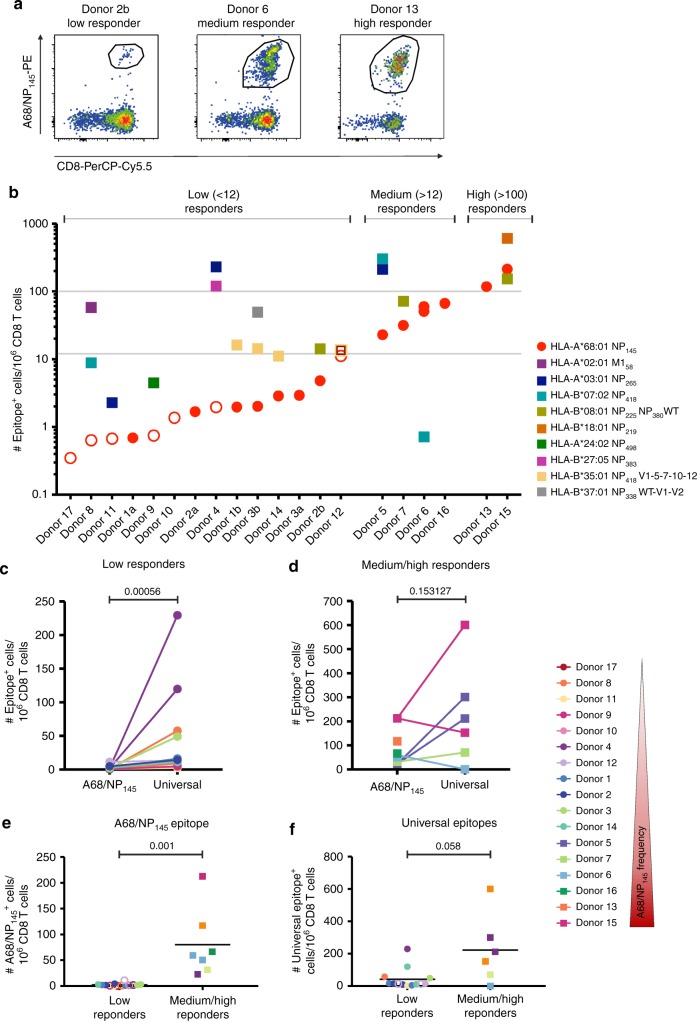
A68/NP_145_-specific CD8^+^ T cell responses vary greatly across the donors. **a** Representative FACS profiles of A68/NP_145_-tetramer staining of CD8^+^ T cells. **b** Frequency of A68/NP_145_^+^ CD8^+^ T cells (red circles) and universal influenza virus-specific CD8^+^ T cells (colored squares). <12 tetramer-specific CD8^+^ T cells/10^6^ CD8^+^ T cells are considered low responses (*n* = 11), >12 tetramer-specific CD8^+^ T cells/10^6^ CD8^+^ T cells medium responses (*n* = 4) and >100 tetramer-specific CD8^+^ T cells/10^6^ CD8^+^ T cells high responses (*n* = 2). Tetramer^+^CD8^+^ T cells detected at <10 cells counted within the tetramer-positive gate are indicated in open symbols (Supplementary Fig. 2). Frequency comparison between A68/NP_145_- vs. universal influenza virus-specific CD8^+^ T cells in low responders (**c**) and medium/high responders (**d**). **e** Frequency comparison of A68/NP_145_^+^CD8^+^ T cells between low and medium/high responders. **f** Frequency comparison of universal epitope-specific CD8^+^ T cells between low and medium/high responders. **c**–**f** Bar indicates mean response of the donors. Exact *p* values are indicated above the graphs. Low responding donors (circles), high responding donors (squares), <10 cells counted (open symbols). Statistical analysis was performed using a Mann–Withney test. Exact *p* value are indicated above the graphs.

To probe the magnitude of established A68/NP_145_^+^CD8^+^ T cell populations, we assessed A68/NP_145_-specific CD8^+^ T cells directly ex vivo using a tetramer-associated magnetic enrichment (TAME)^43,45^ in 17 healthy HLA-A*68:01-expressing individuals (Fig. 3a, b, Supplementary Fig. 1b, Table 1). Frequencies of A68/NP_145_^+^CD8^+^ T cells, calculated relative to total CD8^+^ T cell numbers in an unenriched fraction^45,46^, were compared with frequencies of influenza virus-specific CD8^+^ T cell responses directed against other well-known prominent universal HLAs (Table 1, in bold)^9,10^ using single- or dual-tetramer enrichments. The frequencies of tetramer-enriched A68/NP_145_^+^CD8^+^ T cells revealed three types of A68/NP_145_-specific CD8^+^ T cell responders (Fig. 3b). Out of 17 individuals, 11 were classified as low responders with <12 A68/NP_145_^+^CD8^+^ T cells/10^6^ CD8^+^ T cells. Within those, seven donors had a total of <10 counted A68/NP_145_^+^CD8^+^ T cells within the whole enriched fraction, which was sufficient for analysis of frequencies but not phenotypes (Supplementary Fig. 2). The remaining six donors (35%) displayed substantial pools of A68/NP_145_^+^CD8^+^ T cells, with four donors being medium responders (>12 A68/NP_145_^+^CD8^+^ T cells/10^6^ CD8^+^ T cells) and two donors being high responders (>100 A68/NP_145_^+^CD8^+^ T cells/10^6^ CD8^+^ T cells) (Fig. 3b).

**Table 1:** Demographics and HLA typing of the donors used in this study.

Donor	Age	Sex	Specifications	HLA-A haplotypes	HLA-B haplotypes	Year of recruitment	A68-NP_145_ Responder
D17	NR	NR	Healthy donor	01:01, **68:01**	08:01, 15:01	NR	Low
D8	30	NR	Healthy adult	**02:01**, **68:01**	**07:02**, 40:01	2014	Low
D11	21	NR	Adenovirus infected	**03:01**, **68:01**	40:01, 56:01	2010	Low
D1	22/25	M	Healthy adult	33:03, **68:01**	**35:01**, 58:01	a2015,b2018	Low
D9	49	NR	Healthy adult	**24:02**, **68:01**	15:18, 40:01	2013	Low
D10	29	NR	Healthy adult	02:01, **68:01**	40:02, 44:02	2014	Low
D2	48/51	M	Healthy adult	01:01, **68:01**	**08:01**, 40:01	a2015,b2018	Low
D4	31	F	Healthy adult	**03:01**, **68:01**	**27:05**, 40:01	2018	Low
D3	42/46	F	Healthy adult	01:01, **68:01**	**37:01**, **35:01**	a2015,b2018	Low
D14	NR	NR	Healthy donor	11:01, **68:01**	14:02, **35:01**	2017	Low
D12	90	M	Viral pneumonia Influenza virus negative	30:02, **68:01**	**18:01**, 35:03	2014	Low
D5	52	M	Healthy adult	**03:01**, **68:01**	**07:02**, 35:03	2014	Medium
D7	74	F	Healthy elderly	01:01, **68:01**	**08:01**, 44:02	2013	Medium
D6	29	M	Healthy adult	33:01, **68:01**	**07:05**, 14:02	2016	Medium
D16	NR	NR	Healthy donor	32:01, **68:01**	14:01, 44:02	NR	Medium
D13	30	NR	Influenza A virus infection	02:06, **68:01**	13:01, 15:18	2010	High
D15	NR	NR	Healthy donor	01:01, **68:01**	**08:01**,**18:01**	2017	High
D18	27	M	Healthy adult	01:01, 68:01	08:01	2018	NT

HLA bold indicates HLA used for TAME

*NR* not reported, *NT* not tested

Strikingly, within the low-responders, A68/NP_145_^+^CD8^+^ T cell pools were subdominant as compared with the frequency of other dominant universal influenza-specific CD8^+^ T cell populations within the same individuals (*p* = 0.00056; Fig. 3b, c). In contrast, the frequencies of A68/NP_145_^+^CD8^+^ T cells within medium and high responders were comparable to the frequencies of CD8^+^ T cells directed at universal influenza epitopes (*p* = 0.153; Fig. 2b, d), indicating the immunodominance potential of A68/NP_145_^+^CD8^+^ T cells in at least some donors. These results clearly demonstrate that the establishment of substantial A68/NP_145_^+^ CD8^+^ T cell populations is far from being uniform across the donors (*p* = 0.001, Fig. 3e). Although there was a trend for a lower overall CD8^+^ T cell frequency directed at the universal influenza epitopes in the low responders, as compared with the medium and high responders, this was not significant (*p* = 0.058, Fig. 3f). In addition, no correlation was found between the frequency of A68/NP_145_^+^CD8^+^ T cells and the frequency of CD8^+^ T cells directed against the universal epitopes (*n* = 13, *R*_s_ = 0.3518, *p* = 0.1397, Supplementary Fig. 3). Including additional donors may further strengthen the trend for an overall lower influenza virus-specific CD8^+^ T cell response in HLA-A*68:01 positive individuals, however, could unfortunately not be confirmed due to the low frequency of HLA-A*68:01 donors in our cohorts.

### Distinct A68/NP_145_CD8^+^ T cell phenotypes across responders

**Fig. 4:**
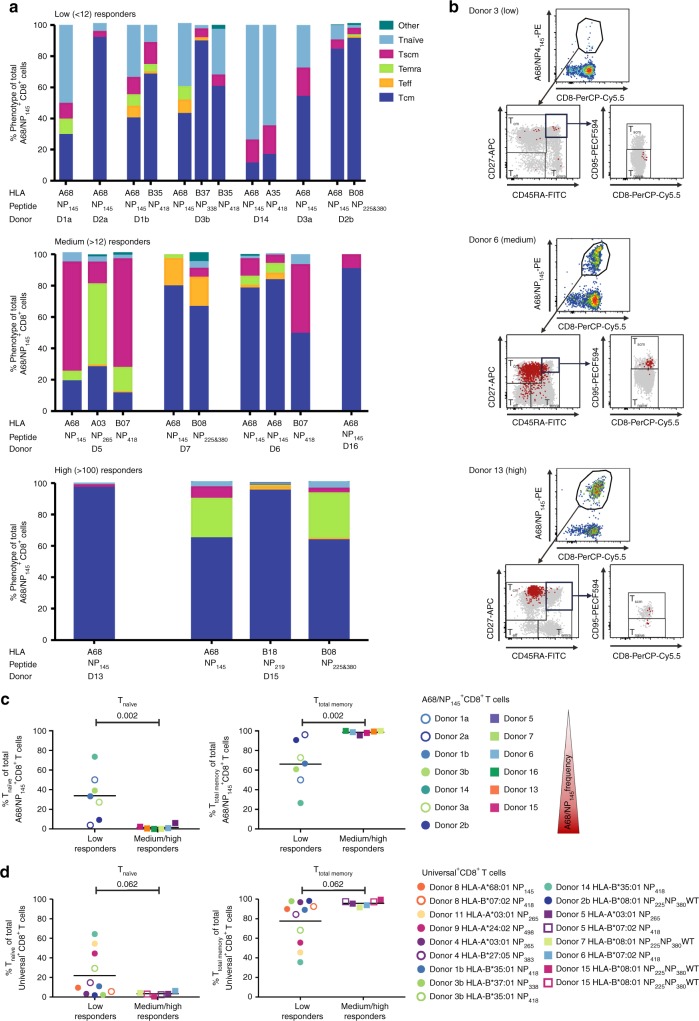
Phenotypes of A68/NP_145_-specific and universal influenza-specific CD8^+^ T cells. **a** Phenotypes of A68/NP_145_- and universal influenza virus-specific CD8^+^ T cells of low (<12 A68/NP_145_-specific CD8^+^ T cells/10^6^ CD8^+^ T cells) (*n* = 4), medium (>12) (*n* = 4), and high (>100) responders (*n* = 2). **b** Representative FACS panels indicate the gating strategy used to characterize A68/NP_145_-specific CD8^+^ T cell response. Representatives for A68/NP_145_-tetramer staining (first panel), CD27, CD45RA staining to identify T_eff_ (CD27^−^CD45RA^−^), T_emra_ (CD27^−^CD45RA^+^), and T_cm_ (CD27^+^CD45RA^−^) cells (second panel), followed by CD95, CD8 staining to identify T_naïve_-like (CD27^+^CD45RA^+^CD95^−^) and T_scm_ (CD27^+^CD45RA^+^CD95^+^) cells (third panel). Gray dots are total CD8^+^ T cells in unenriched sample, red dots are A68/NP_145_^+^CD8^+^ T cells in enriched sample. **c** Frequency comparison of *T*_naïve_ and T_total memory_ (T_cm_ + T_em_ + T_emra_ + T_scm_) A68/NP_145_^+^CD8^+^ T cells in low vs. medium/high responders. We included two measurements for donor 1, 2, and 3: open symbols were used for samples collected in 2015, closed symbols for samples collected in 2018 (see also Table 1). **d** Frequency comparison of T_naïve_-like and T_total memory_ universal-specific CD8^+^ T cells in low vs. medium/high responders. **c**–**d** Bar indicates mean response of the donors. Statistical analysis was performed using a Mann–Withney test. Exact *p* value are indicated above the graphs.

To understand whether low detection of A68/NP_145_^+^CD8^+^ T cell populations found in 65% of our donors resulted from the establishment of differential naïve/memory subsets within the A68/NP_145_^+^ CD8^+^ T cells, we compared directly ex vivo the phenotypes of A68/NP_145_^+^CD8^+^ T cells both (i) across HLA-A*68:01-expressing donors, and (ii) with phenotypes of CD8^+^ T cells directed at universal influenza epitopes (Fig. 4). We used three surface markers, CD27, CD45RA, and CD95 to delineate naïve-like (T_naïve_, CD27^+^CD45RA^+^CD95^−^), effector/effector memory (T_eff_, CD27^−^CD45RA^−^), terminally differentiated effector (T_emra_, CD27^−^CD45RA^+^), central memory (T_cm_, CD27^+^CD45RA^−^) and stem cell memory (T_scm_, CD27^+^CD45RA^+^CD95^+^) CD8^+^ T cell subsets in low, medium, and high responding donors (Fig. 4a, b, Supplementary Fig. 1b).

In three out of four low-responding donors, a markedly higher proportion of the A68/NP_145_^+^CD8^+^ T cells displayed a naïve-like phenotype (mean 33.77% ± 23.83; *n* = 4 donors), when compared with A68/NP_145_^+^CD8^+^ T cells in medium/high responders (mean 1.52% ± 2.17; *n* = 6 donors; *p* = 0.002) (Fig. 4c). In contrast, the proportion of total memory A68/NP_145_^+^CD8^+^ T cells (T_cm_, T_emra_, T_eff_, T_scm_) (T_total memory_) in medium/high responders (mean 98.59% ± 1.7; *n* = 6 donors) was significantly higher compared with low responders (66.23% ± , 23.84, *n* = 4 donors; *p* = 0.002) (Fig. 4c), demonstrating a switch from naïve-like to memory phenotypes in A68/NP_145_^+^CD8^+^ T cells within medium/high responding donors.

Phenotypic analysis of the universal influenza-specific CD8^+^ T cell populations revealed no significant differences within the naïve-like (*p* = 0.062) or memory (*p* = 0.062) phenotypes when low and medium/high responders were compared (Fig. 4d), consistent with their similar response magnitudes. Detection of memory phenotypes within all the universal CD8^+^ T cell sets also verifies that our A68^+^ donors had previous influenza encounters and thus their A68/NP_145_^+^ CD8^+^ T cells were exposed to the antigenic stimulation during previous influenza virus infection across all the low, medium, and high responding donors.

### Diverse TCRαβ repertoires within A68/NP_145_^+^CD8^+^ T cell pools

**Fig. 5:**
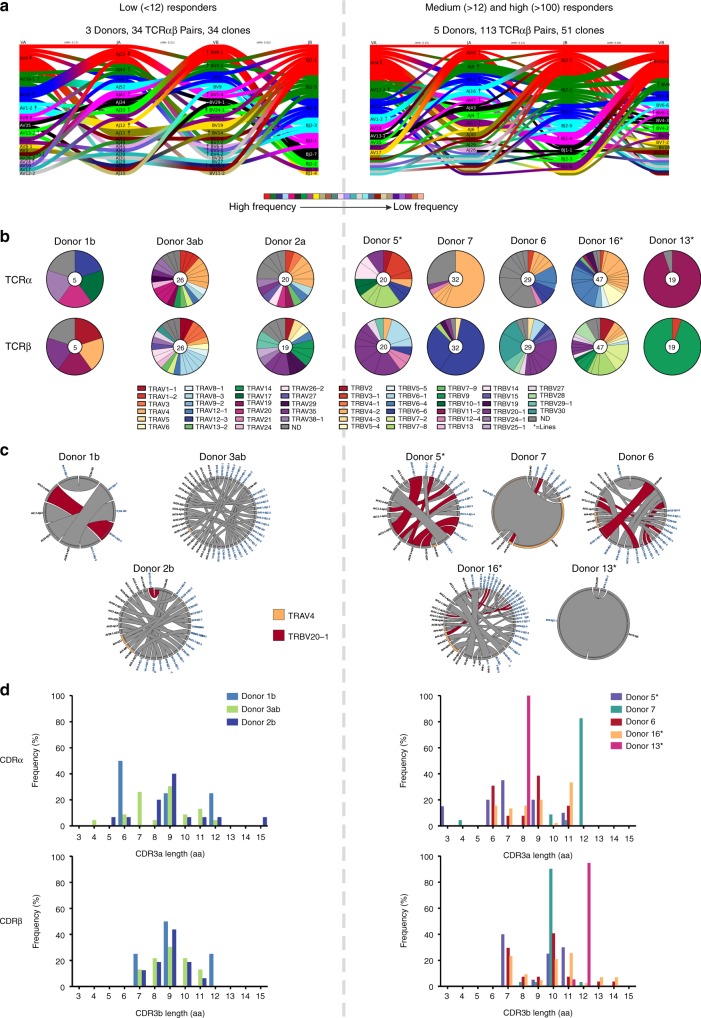
High clonal diversity in the A68/NP_145_-specific TCR repertoire. **a** Gene segment usage and pairing landscape are shown for low (*n* = 3) and medium/high (*n* = 5) responders. Each clonotype is assigned the same vertical length irrespective of clonotype size. Each vertical stack reflects V and J gene segment usage and pairing is shown by curved connecting lines. Genes are colored by frequency of distribution. Enrichment or depletion of gene usage is indicated by up or down arrows respectively where one arrowhead correlates to a twofold increase or decrease. **b** Pie charts of TRAV and TRBV gene usage in individual donors. Total number of analyzed gene sequences are indicated within the pie charts. **c** Circos plots showing the distribution of TRA-TRB paired clonotypes across donors. Each segment defines an individual clonotype and width of the segment correlates to the frequency of the clonotype. Private clonotypes are in gray. The commonly observed clonotypes shared between different donors are shown in red (TRBV20-1) and orange (TRAV4). **d** Distribution of CDR3α and CDRβ amino acid lengths in low and medium/high responding donors. An asterisk indicates that TCR clonotypes were established on in vitro expanded T cell lines.

To understand differential A68/NP_145_^+^ CD8^+^ T cell responses across HLA-A*68:01 donors, we dissected the clonal diversity and composition within A68/NP_145_^+^ CD8^+^ T cells found in low- and medium/high-responders. We used a combination of single-cell index sorting and multiplex RT-PCR^43,47^ to amplify both TCRα and TCRβ chains within a single cell, enabling analysis of the paired A68/NP_145_^+^CD8^+^ TCRαβ repertoire in three low-responders and five medium/high-responders (Fig. 5, Table 2, Supplementary Table 3).

**Table 2:** Frequencies of paired TRBV-TRBJ/TRAV-TRAJ expanded clonotypes within A68/NP_145_ TCRs in human PBMCs.

Clone ID	TRBV	TRBJ	CDR3b	CDR3β length	TRAV	TRAJ	CDR3a	CDR3α length	Low				Medium				High
									D1b	D3b	D3a	D2b	D5^a^	D7	D6	D16^a^	D13^a^
AF	TRBV20-1	TRBJ1-1	CSAETGNTEAFF	7	–	–	–	–				10					
AY	TRBV20-1	TRBJ2-1	CSADNVAGGPGSEQFF	11	TRAV13-1	TRAJ43	CATYDMRF	3					15				
AZ	TRBV6-1	TRBJ2-1	CASSEPRDEQFF	7	TRAV1-2	TRAJ9	CAVETGGFKTIF	7					15				
BA	TRBV20-1	TRBJ2-7	CSASQDPYEQYF	7	TRAV35	TRAJ37	CAGQTTSNTGKLIF	9					10				
BB	TRBV20-1	TRBJ2-1	CSALYPLAGPGNEQFF	11	TRAV26-2	TRAJ30	CILNRDDKIIF	6					10				
BM	TRBV6-6	TRBJ2-1	CASSSPSGVYNEQFF	10	TRAV4	TRAJ4	CLVGDLINSGGYNKLIF	12						56			
BN	TRBV6-6	TRBJ2-1	CASSSPSGVYNEQFF	10	–	–	–	–						28			
BT	TRBV30	TRBJ2-7	CAWSPAGLAMYEQYF	10	–	–	–	–							17		
BU	TRBV20-1	TRBJ1-1	CSAESGNTEAFF	7	–	–	–	–							14		
BV	TRBV30	TRBJ2-7	CAWSPAGLAMYEQYF	10	TRAV12-1	TRAJ29	CVVNANSGNTPLVF	9							10		
BW	TRBV6-1	TRBJ2-7	CASSEAGGPGYEQYF	10	–	–	–	–							7		
BX	TRBV29-1	TRBJ1-4	CSVRDISTNEKLFF	9	–	–	–	–							7		
BY	TRBV20-1	TRBJ1-6	CSAEDGNSPLHF	7	TRAV12-2	TRAJ47	CAVKYGNKLVF	6							7		
CK	TRBV7-8	TRBJ2-2	CASSDSAGELFF	7	TRAV6	TRAJ4	CALSGYNKLIF	6								11	
CL	TRBV7-2	TRBJ2-5	CASSSIGVAGEETQYF	11	TRAV12-1	TRAJ11	CVVNVLLNSGYSTLTF	11								11	
CM	TRBV2	TRBJ2-1	CASNDPPGATNNEQFF	11	TRAV4	TRAJ42	CLVGGGSQGNLIF	8								9	
CN	TRBV28	TRBJ1-1	CASSSLIQANTEAFF	10	TRAV12-1	TRAJ42	CVVNVGYGGSQGNLIF	11								9	
CO	TRBV7-2	TRBJ2-7	CASSDLAGTSGTNTYEQYF	14	TRAV4	TRAJ21	CLVGGNFNKFYF	7								6	
CP	–	–	–	–	TRAV12-1	TRAJ11	CVVNRVENSGYSTLTF	11								6	
CQ	TRBV7-8	TRBJ2-2	CASSDSAGELFF	7	TRAV8-1	TRAJ6	CAANSGGSYIPTF	8								4	
CR	TRBV4-2	TRBJ2-5	CASSQVGTTLETQYF	10	TRAV29	TRAJ29	CAASAISGNTPLVF	9								4	
CS	TRBV9	TRBJ2-6	CASSVNPAQGSGANVLTF	13	TRAV9-2	TRAJ34	CALIYNTDKLIF	7								4	
DK	TRBV9	TRBJ2-1	CASSVDLKAGEEGEQFF	12	TRAV19	TRAJ9	CALSNTGGFKTIF	8									95
% expanded αβ pairs of total TCRαβ repertoire									0	0	0	10	50	84	62	64	95
Total number αβ pairs									5	4	22	20	20	32	29	47	19

CDR3 length was calculated based on underlined CDR3 sequence*D* donor

^a^Indicates that TCR clonotypes were established on T cell lines

In contrast to the narrowed/skewed TCR repertoires directed at the majority of previously reported long peptide/HLA complexes^26–31^, the A68/NP_145_^+^CD8^+^ TCRαβ repertoires utilized a broad array of TRBV (T receptor β variable) and TRAV (T receptor α variable) gene segments in low-responders and medium/high responders (Fig. 5a, Table 2, Supplementary Table 3). The most common gene segments were TRBV20-1 and TRAV4 observed in six out of eight donors (Fig. 5b, c). Interestingly, donor 7 (medium responder) and 13 (high responder) expressed a highly restricted private TRAV and TRBV combinations, namely TRBV6-6/TRAV4 and TRBV9/TRAV19, respectively (Fig. 5b, c).

Further dissection of the CDR3αβ clonotypic signatures revealed a lack of common motifs within the individual donors (Table 2, Supplementary Table 3) and absence of a shared CDR3αβ signature (public clonotypes) across HLA-A*68:01-expressing donors. Both low and medium/high responders displayed large variation in the length of the CDR3α loop ranging from 4 to 15 aa and 3 to 12 aa, respectively (Fig. 5d). Similarly, the length of the CDR3β loop was variable, ranging from 7 to 12 aa in low-responders and 7 to 14 aa in medium/high responders (Fig. 5d).

Overall, the A68/NP_145_^+^CD8^+^ TCRαβ repertoire was strikingly diverse, with no common features shared between donors. Thus, the A68/NP_145_^+^CD8^+^ T cell response does not seem to be limited by the availability of particular TCRαβs that can recognize the long and flexible 12 aa NP_145_ peptide in the context of HLA-A*68:01.

### Expanded A68/NP_145_^+^TCRαβ clones in medium/high responders

**Fig. 6:**
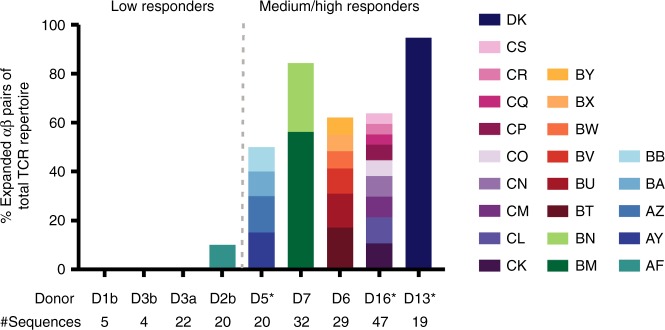
Level of expanded TRBV-TRBJ/TRAV-TRAJ clonotypes. Frequency of expanded TRBV-TRBJ/TRAV-TRAJ clonotypes observed in the total sequenced TCRαβ repertoire within each donor (*n* = 8). Letters in legend correspond with clone ID in Table 2. Total number of sequences within each donor is indicated below the graph. An asterisk indicates that TCR clonotypes were established on in vitro expanded T cell lines.

Despite A68/NP_145_^+^CD8^+^ TCRαβ repertoire diversity in all the low and medium/high responders, it became evident that the A68/NP_145_^+^CD8^+^ TCRαβ repertoires within medium/high responders contained a high proportion (*n* = 5, mean 71%, range 50–95%) of expanded TCRαβ clonotypes as compared with low responders (*n* = 3, mean 2.5%, range 0–10%) (*p* = 0.016) (Fig. 6, Table 2). Such high proportion of the expanded TCRαβ clonotypes within medium/high responders provides clear evidence of a correlation between large clonal expansions and immunodominance observed in medium and high A68/NP_145_^+^CD8^+^ T cell responders. This further suggests that minimal clonal expansions underlie poor pre-existing memory A68/NP_145_^+^CD8^+^ T cell pools in low- and non-responders. Thus, subsequent boosting of A68/NP_145_^+^CD8^+^ T cells in low responders might be one of the ways to ensure HLA-A*68:01-expressing individuals have substantial numbers of memory A68/NP_145_^+^CD8^+^ T cells and therefore at least some level of protection against unpredicted newly-emerged influenza viruses.

### Memory phenotypes associated with high frequency TCRαβs

**Fig. 7:**
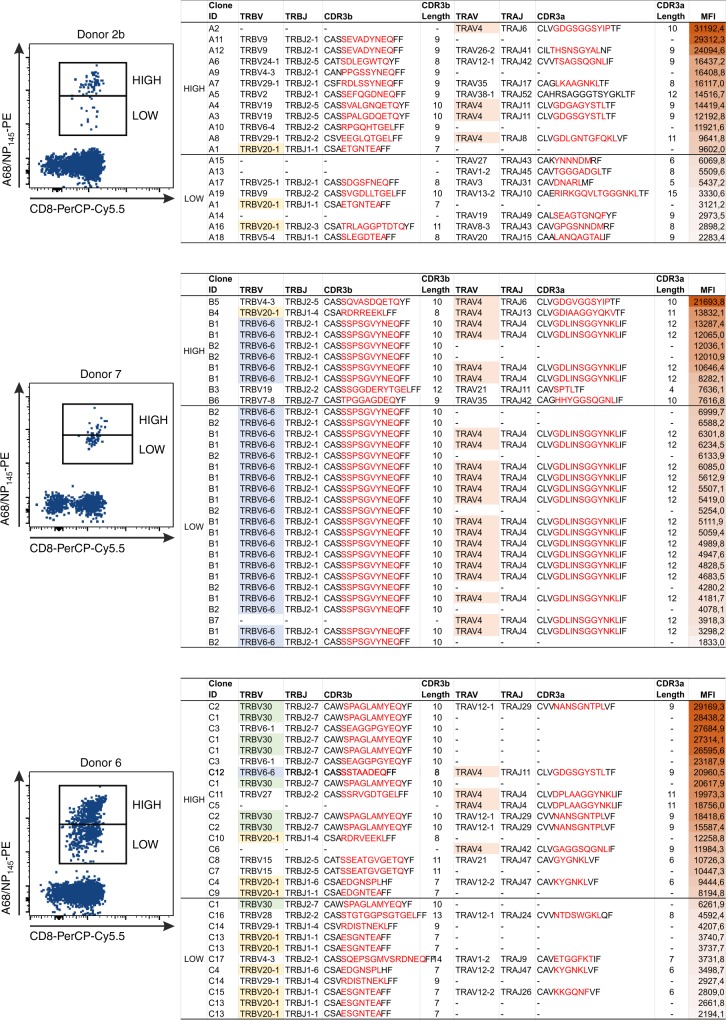
TCRαβ clonotype distribution based on A68/NP_145_-tetramer avidity. FACS panels (left) show gating of high and low avidity A68/NP_145_^+^ CD8^+^ T cells of low (donor 2b) and medium (donor 7 and 6) responders. Tables show clonotypes arranged based on A68/NP_145_–tetramer avidity (right). Last column in the table shows MFI of individual clonotypes, MFI gradient color from high MFI (dark red) to low MFI (white). TRBV20-1 (yellow) and TRAV4 (orange) are commonly observed clonotypes that are shared between donors, whereas TRBV6-6 (blue) and TRBV30 (green) are donor-specific clonotypes observed at high frequency.

As our experiments used single-cell index sorting, we were in a position to directly link the individual A68/NP_145_^+^CD8^+^ TCRαβ clonotypes to the exact phenotype of each analysed TCRαβ. We were interested to link the A68/NP_145_^+^CD8^+^ TCRαβ repertoires with their respective HLA-A*68:01-NP_145_ tetramer avidity, as previous reports suggested that HLA-A*68:01-specific TCRs require high affinity binding to the peptide-HLA complex (pHLA)^48^ to overcome reduced affinity for the CD8 binding site due to a polymorphism at position 245 (A245V) within HLA-A*68:01^49^. Before the single-cell index sort technique was available, the high and low avidity populations of A68/NP_145_^+^CD8^+^ T cells within donor 16 were directly single-cell sorted into two individual plates, which revealed two distinct TCR repertoires between the high- and low-avidity populations (Supplementary Fig. 4). With the use of index sorting, we were able to further discriminate between high and low avidity A68/NP_145_^+^CD8^+^ T cells based on their actual tetramer mean fluorescence intensity (MFI) (Fig. 7).

Of all index-sorted donors (*n* = 5; 1b, 2b, 3b, 6, and 7), A68/NP_145_^+^CD8^+^ T cell responses within donors 2b, 6, and 7 (*n* = 3) were sufficient enough to discriminate between high- and low-avidity populations and therefore to establish any direct links between TCRαβ repertoires and their respective MFI (Fig. 7). When focusing our analysis on the two major gene segments observed in six out of eight donors (TRBV20-1 and TRAV4) (Table 2, Supplementary Table 3), we found that TRBV20-1 was more prevalent in the low avidity populations (59% of the TRBV20-1 clonotypes across all four donors), whereas the TRAV4 was more common in the high avidity populations (54% of the TRAV4 clonotypes across all four donors) (Fig. 7, Supplementary Fig. 4). Clonotype TRBV30 was only observed in donor 6 and was more prevalent in the high avidity population (88% of the TRBV30 clonotypes) (Fig. 7). Interestingly, in donor 7 the majority of the TCRαβ repertoire comprises of one expanded TCRαβ clonotype TRBV6-6-CASSSPSGVYNEQ and TRAV4^−^CLVGDLINSGGYNKLIF, and a number of smaller clonotypes (Fig. 7). The dominant clonotype was found across higher and lower MFIs. This indicates that MFI of tetramer binding might be not only affected by TCRαβ chains but also most probably TCR levels, TCR dynamics, TCR spatial arrangements, and/or other intrinsic factors.

**Fig. 8:**
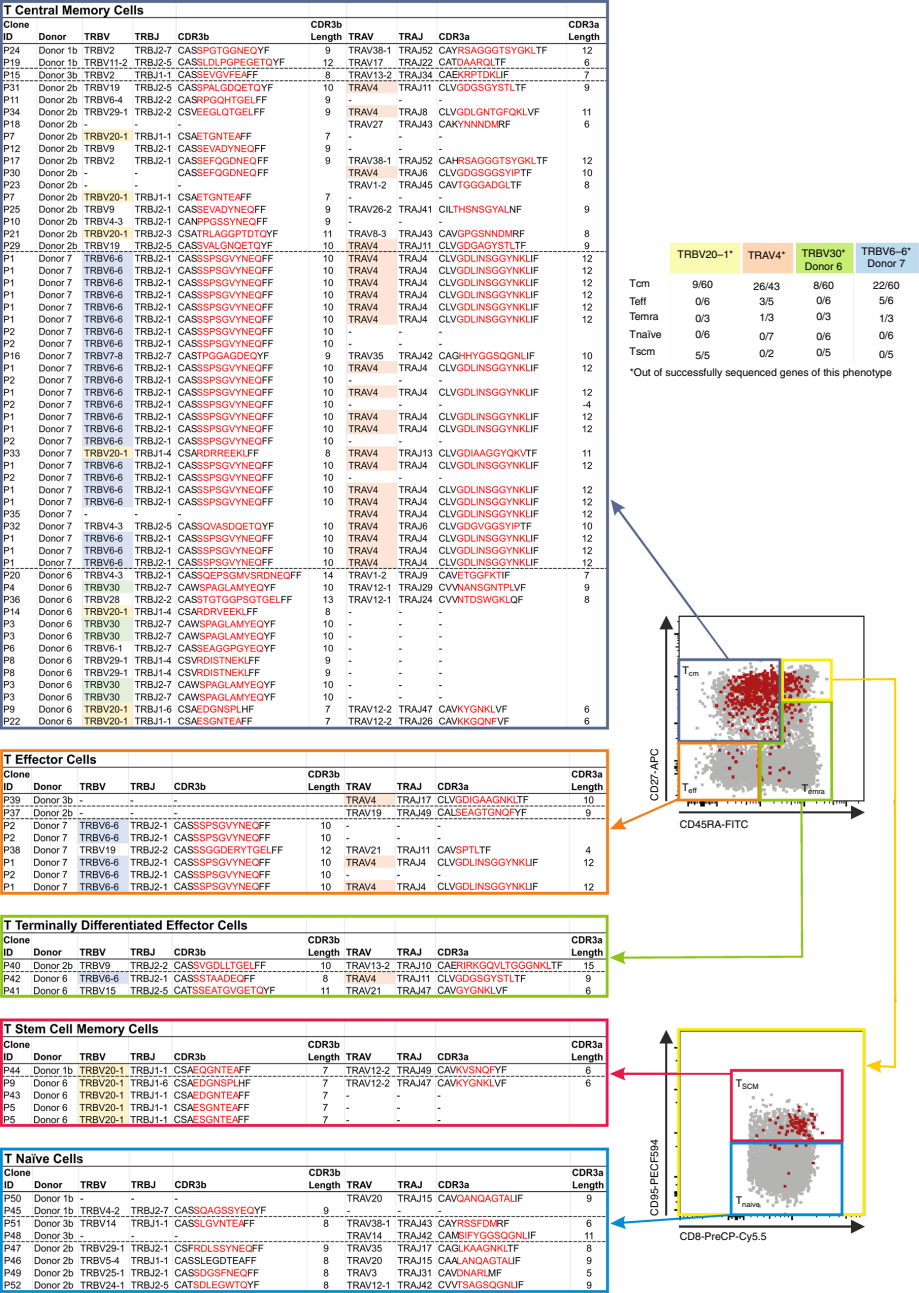
TCRαβ clonotype distribution based on their individual phenotype. FACS panels (center) indicate the gating strategy used to define T_eff_ (CD27^−^CD45RA^−^), T_emra_ (CD27^−^CD45RA^+^), and T_cm_ (CD27^+^CD45RA^−^) cells (top FACS plot) and T_naïve_-like (CD27^+^CD45RA^+^CD95^−^) and T_scm_ (CD27^+^CD45RA^+^CD95^+^) cells (bottom FACS plot). Gray dots are total CD8^+^ T cells in unenriched sample, red dots are A68/NP_145_^+^CD8^+^ T cells. Clonotypes of A68/NP_145_^+^CD8^+^ T cells are listed based on phenotype (*n* = 5 donors). TRBV20-1 (yellow) and TRAV4 (orange) are commonly observed clonotypes, which are shared between donors whereas TRBV6-6 (blue) and TRBV30 (green) are donor-specific clonotypes observed at high frequency.

Subsequently, we dissected the A68/NP_145_^+^CD8^+^ TCRαβ repertoires of all index-sorted donors (*n* = 5; 1b, 2b, 3b, 6, and 7) according to their matched phenotypes (Fig. 8). Here, we focused on the commonly observed clonotypes TRBV20-1 and TRAV4 (shared between donors 1b, 2b, 3b, 5, 6, 7, and 16) and the donor-specific clonotypes TRBV30 (donor 6) and TRBV6-6 (donor 7), which were detected at relatively high frequencies in those donors. We found that the expanded TCRαβs within common gene segments (TRBV20-1 and TRAV4) and the high frequency individual clonotypes (TRBV30 and TRBV6-6) were highly prevalent in the memory CD8^+^ T cell populations (Fig. 8). These results confirm that the large TCRαβ clonal expansions observed within the medium and high HLA-A*68:01-responding donors were predominantly of the memory phenotype (Figs. 6 and 8).

Overall, our results indicate that even though the A68/NP_145_^+^CD8^+^ TCRαβ repertoire is highly diverse, with preferred TCRαβ gene usage in certain individuals, including TRBV20-1, TRAV4, TRBV30, and TRBV6-6, largely-expanded clonotypes displaying a memory phenotype underlie greater A68/NP_145_^+^CD8^+^ T cell response magnitude in medium to high responders.

## Discussion

In this study, we dissected the immune response against the 12 aa NP_145_ peptide presented by an influenza risk-associated HLA-A*68:01 molecule. We found that the NP_145_ viral peptide was highly conserved across influenza strains, except for position 146 (P2 anchor residue position of the peptide), although this did not result in viral escape from the A68/NP_145_-specific CD8^+^ T cell response. The HLA-A*68:01-NP_145_ crystal structure revealed that NP_145_ peptide is highly mobile in the cleft of the HLA-A*68:01 molecule. The low frequencies of A68/NP_145_^+^CD8^+^ T cells in 65% HLA-A*68:01 donors, combined with their large naïve-like populations and non-expanded TCRαβ clonotypes, indicate that A68/NP_145_^+^CD8^+^ T cells might be difficult to recruit during influenza virus infections. Conversely, largely-expanded TCRαβ clonotypes were commonly observed in memory CD8^+^ T_cm_ populations in medium/high responders, suggesting that it might take several influenza exposures, thus repeated A68/NP_145_^+^CD8^+^ T cell boosting, to establish pre-existing immunodominant A68/NP_145_^+^CD8^+^ T cell memory pools.

Donors used to study the cross-reactivity of the A68/NP_145_-specific CD8^+^ T cell response were likely to have been exposed to viruses expressing both the 146A and 146T variant of the NP_145_ peptide. Donor 6 was born in 1987, donor 18 was born in 1991. Although the exact date of birth for donor 15 is unknown, the donor was recruited in 2017 and would have been 18 years or older at time of recruitment, hence born before 1999. It was demonstrated that by the age of 3, 80% of the children would have experienced at least one IAV infection, increasing to 100% by the age of 7^50^. Thus, all three donors would have been infected with an IAV expressing the 146T variant of the peptide, which was expressed in A/H3N2 and A/H1N1 strains circulating prior to 2001. Even though influenza virus infection is less frequently observed in adults than in children, adults still encounter two influenza virus infections per decade^51^. It is therefore reasonable to assume that all three donors would have had at least one additional influenza virus infections after 2001 with either the A/H3N2 virus strain and/or the A/H1N1pdm09 strain, both expressing the 146A variant of the peptide. The chance that these donors would have encountered the 146V variant of the peptide via natural infection is highly unlikely, as this variant was only observed in seven out of the 24408 human IAV isolates recorded between 1918 and 2018.

Even though rapid fixation of amino acids substitutions inside CD8^+^ T cell epitopes, especially at anchor or TCRαβ binding residues, are often associated with immune escape^15,52^, this was not the case for the rapid fixation of A146T and T146A substitutions in NP proteins of human influenza viruses. Both alanine and threonine have been shown to bind in the HLA-A*68:01 binding cleft in a similar fashion. Furthermore, when we expanded A68/NP_145_^+^CD8^+^ T cells from three independent donors using the 146A or 146T variants of the NP_145_ peptide, both variants expanded A68/NP_145_^+^CD8^+^ T cell populations and following restimulation responded to all three variants of the NP_145_ peptide (146A, 146T, and 146V, respectively), suggesting high cross-reactivity between NP_145_ variants. However, according to the IEDB database (www.iedb.org), the NP_145–156_ peptide overlaps with at least four other peptides, namely NP_146–154_ (HLA-A*02:03, 68:02, HLA-B*14:02, 02:02, and HLA-C*06:02), NP_140–148_ (HLA-A*01:01,26:01, 30:02, 80:01, HLA-B*15:01, 15:17, 35:01, 57:01, 58:01), NP_140–150_ (HLA-B*15:01) and NP_139–156_ (HLA-B*15:01). It is thus possible that the variation observed at position NP_146_ is driven by the virus’ ability to escape from CD8^+^ T cells responses directed against one or more of these overlapping peptides instead. We solved the structure of the HLA-A*68:01-NP_145_ with an Alanine at position 146 (P2-Ala) as this variant was present in both A/H3N2 and A/H1N1 influenza viruses that circulated in the last decade. We observed that the P2-Ala is well-accommodated within the antigen-binding cleft of HLA-A*68:01, and that the long peptide is highly mobile as previously observed for other HLA-A*68:01-restricted peptides^53^. This is in accordance with a previously solved binary structure of HLA-A*68:01 and a 9 aa NP_91–99_ peptide showing that the HLA-A*68:01-bound peptide was highly flexible, thus allowing the binding of overlapping peptides of different lengths but of conserved residues located at P2 and PΩ^53^. In addition, HLA-A*68:01 can also present peptides of canonical lengths (9–10 mer), such as the 9 aa RT313 peptide from HIV^53^, or a 10 aa self-peptide with a C-terminal extension that bulges out of the cleft^54^, both adopting a rigid conformation in the cleft of the HLA-A*68:01 molecule.

Approximately 30 structures of unique MHC class I-long peptide (≥11 aa) complexes have been structurally solved, where 2/3 displayed a rigid conformation^19^. TCR repertoires directed at MHC class I-restricted long peptides, were observed to have a highly biased TCR gene usage^26–31^. These observations might suggest that TCRs have a limited capacity to engage with such long peptide-HLA-I complexes, which may limit the recruitment of peptide-specific CD8^+^ T cells during infection. This was also observed in our donors where low frequencies of A68/NP_145_^+^CD8^+^ T cells were observed in 65% HLA-A*68:01 donors tested. A substantial proportion of the A68/NP_145_^+^CD8^+^ T cells in three out of four low-responding donors displayed a naïve-like phenotype and single (non-expanded) TCRαβ signatures. Interestingly, the TCRαβ repertoire was highly diverse in six out of eight HLA-A*68:01 donors, including all three low responding donors. Only two other long peptides, the tumor antigen HLA-B*07:02/NY-ESO-1 (13 aa) and HIV-p24 HLA-B*57:03/KF11 (11 aa), have been described to display a more diverse TCRαβ repertoire^20,30,34^. In alignment with a study from Chan et al., we observed variability in CDR3 length without a consensus sequence motif^20^. One possible explanation is that flexible long peptides could result in multiple and potentially suboptimal TCRαβ binding sites, leading to a more diverse TCRαβ repertoire^20,32,33^. However, the low frequency of A68/NP_145_^+^CD8^+^ T cells in combination with a high variety of TCRαβ clonotypes and less frequent TCRαβ gene segments expressing a naïve-like phenotype, indicates that the high flexibility of this epitope might possibly prevent the effective recruitment of CD8^+^ T cells during influenza virus infections, thus might need to be primed by rationally-designed T cell vaccines.

It is unlikely that the ineffective recruitment of A68/NP_145_^+^CD8^+^ T cells is the result of the reduced CD8 binding affinity of the HLA-A*68:01 molecule^49^. Previous studies have shown that the CD8 co-receptor maintained the majority of its biological activity, even at extremely low binding affinities^55^. Furthermore, the reduced CD8 binding affinity did not affect the functional activity of HLA-A*68:01-NP_89–101_ and HIV_Tat_-specific CD8^+^ T cells^48,55^. In addition, it was shown that HLA-A*68:01-HIV_Tat_-specific TCR binding was within the normal TCR-pHLA binding range^56^, which was consistent with our tetramer-avidity data.

Overall, A68/NP_145_^+^CD8^+^ T cells have an immunodominant memory potential, as detected in 35% of our donors. However, as the remaining 65% of HLA-A*68:01-expressing donors had low precursor frequency of A68/NP_145_^+^CD8^+^ T cells, which indicates possible difficulties with the recruitment of A68/NP_145_^+^CD8^+^ TCRαβ clonotypes during influenza-specific responses, as compared with the engagement of TCRαβ clones against universal influenza CD8^+^ T cell epitopes in the same donors. A potential low CD8^+^ T cell frequency against this one NP_145_/HLA-A*68:01 epitope may not greatly affect the disease severity in individuals with additional HLAs capable of presenting universal influenza epitopes mounting robust influenza CD8^+^ T cell responses against these universal epitopes. However, as the frequency of the HLA-A*68:01 allomorph is especially high among the Indigenous populations globally including Southern America (http://www.allelefrequencies.net) and Australia^57^. These Indigenous populations often lack HLA allomorphs that present universal influenza epitopes and thus might lack influenza virus-specific CD8^+^ T cell responses toward other HLAs^9^. The fact that the A68/NP_145_-specific CD8^+^ T cells have an immunodominance potential makes them an interesting target to stimulate by novel CD8^+^ T cell-inducing influenza vaccines. Future research is needed to understand whether their influenza virus-specific CD8^+^ T cell response will benefit from repeatedly boosting, for example by novel CD8^+^ T cell-inducing influenza vaccines.

## Methods

### Human blood samples

Our study assessed influenza-specific CD8^+^ T cell responses in 18 HLA-A*68:01-expressing donors. These 18 donors represent all of our HLA-A*68:01 individuals recruited and HLA typed across six different HLA-typed cohorts, consisting of a total of ∼500 donors. Peripheral blood mononuclear cells (PBMCs) were obtained from donors recruited through the University of Melbourne (UoM), Australian Red Cross Lifeblood (ARCL) (Melbourne), Deepdene Medical Clinic (DMC) (Melbourne), Menzies School of Health Research, Royal Melbourne Hospital (RMH) and the Alfred Hospital (AH) (Table 1). Ficoll-Paque (GE Health-care, Uppsala, Sweden) gradient centrifugation was used to isolate PBMCs, which were subsequently cryopreserved in liquid N_2_ until required. A68/NP_145_^+^CD8^+^ T cell responses for donors 1, 2, and 3 were accessed at two separate time-points, in 2015 and 2018 (Table 1). HLA class I and class II molecular genotyping was performed from genomic DNA by the ARCL. Human experimental work was conducted according to the Declaration of Helsinki principles and approved by the Human Research Ethics Committee (HREC) of the University of Melbourne (Ethics ID #1443389.4), Northern Territory Department of Health and Menzies School of Health Research (ID #HREC-2012-1928) for LIFT donors^57^ and HREC of Monash Health and Melbourne Health (ID #HREC/15/MonH/64) for RMH donors and Alfred Hospital (ID #280/14) for AH donors. All donors provided informed written consent.

The vaccination and infection history of the donors were predominantly unknown. However, it is important to note that the current inactivated influenza vaccine does not induce influenza-specific CD8^+^ T cells responses^4^, thus recent influenza immunisation would not affect CD8^+^ T cell responses tested in this study.

### Protein expression, purification, and crystallization

Soluble class I heterodimers of HLA-A*68:01-containing NP_145_ peptide were prepared by expressing the heavy chain and the β2-microglobulin proteins separately as inclusion body in a BL21 *E. Coli* strain. After several washes, the inclusion bodies were solubilized in 6 M guanidine before being use for refold. The refolding buffer contained 0.1 M Tris-HCl pH8, 2 mM EDTA, 400 mM L-Arginine-HCl, 0.5 and 5 mM Glutathione oxidized and reduced, respectively. Into the chilled refolding buffer was added 90 mg of heavy chain inclusion bodies; 20 mg of β2 m inclusion bodies, and 10 mg of the NP_145_ peptide (purchased from GLbiochem) dissolved in 400 μL of DMSO. After 3 days, the protein was dialyzed and purified using anion exchange and size exclusion columns. Crystals of the HLA-A*68:01-NP_145_ grew at 2.5 mg/ml in 8–14% v/w PEG3350, 0.1 M NaCl, 0.1 M Hepes pH 7.4, 20 mM MgCl_2_, and 5 mM CdCl_2_. The crystals were soaked into a cryoprotectant solution containing the mother liquor solution enriched at 25% v/w PEG3350, and flash frozen in liquid nitrogen. Data were collected on the MX2 beamline^58^ at the Australian Synchrotron, Clayton using an ADSC 315r CCD detector (at 100 K). Diffraction data were processed using XDS software^59^, and scaled with SCALA software^60^ from the CCP4 suite^61^. The structure of HLA-A*68:01-NP_145_ complex was solved by molecular replacement using PHASER (S0907444901012471) with the previously solved structure of HLA-A*68:01 as model (PDB accession number 4HWZ^62^) without the bound peptide. The model was refined with Buster software^63^ after multiple manual model building run to fit the NP_145_ peptide in the structure using Coot software^64^. The final model has been validated using the Protein Data Base validation website, final refinement statistics are summarized in Supplementary Table 1. All molecular graphics representations were created using MacPyMOL v1.7.6.3^65^.

### Viral sequence analysis

To assess the frequency of amino acid variations in the NP_145_ peptide in human A/H1N1 (1918–1957, 1977−2009, and 2009–2018), A/H2N2 (1957–1968), A/H3N2 (1968–2018), H5N1 (1997–2014) and H7N9 (2013–2017) viruses, all full-length NP amino acid sequences available in the influenza virus resource database of the National Center for Biotechnology Information (NCBI; http://www.ncbi.nlm.nih.gov/genomes/FLU), as of 31 July 2018, were downloaded. Sequences with large deletions were excluded using BioEdit, the remaining dataset was analyzed in Ugene 1.16.1 (http://ugene.unipro.ru; Unipro, Novosibirsk, Russia) to assess the frequency of variations at different positions in the NP_145_ peptide. Viruses were analyzed in Excel to determine whether observed frequencies were the result of cluster formation and whether certain mutations became fixed in time.

### Peptides and tetramers

Variants of the IAV NP_145_ peptide (DATYQRTRALVR, DTTYQRTRALVR, and DVTYQRTRALVR) were purchased from GenScript (Piscataway, NJ, USA). HLA^−^A*68:01-NP_145_ (DATYQRTRALVR) monomers were generated in house by refolding each peptide with its restricted HLA α-heavy chain-BirA and β2-microglobulin^66^ before 8:1 conjugation with PE-streptavidin or APC-streptavidin (BD Biosciences, San Jose, CA, USA) to form tetramers.

### Generation of T cell lines

To amplify influenza virus-specific CD8^+^ T cells directed at the HLA-A*68:01-restricted NP_145_ epitope, autologous PBMCs (~3 × 10^6^ cells) from HLA-A*68:01 donors were pulsed with 10 μM NP_145_ peptide (DATYQRTRALVR, DTTYQRTRALVR or pool of DATYQRTRALVR, DTTYQRTRALVR, and DVTYQRTRALVR) in 1 ml serum-free RPMI1640 medium (Invitrogen) for 90 min at 37°C and washed with RPMI. Peptide-pulsed PBMCs were then incubated with autologous nonpeptide-pulsed PBMCs (6 × 10^6^ cells) and cultured for 10 days in cRPMI (RPMI supplemented with 2 mM L-glutamine (Gibco), 1 mM MEM sodium pyruvate (Gibco), 100 µM MEM non-essential amino acids (Gibco), 5 mM HEPES buffer solution Gibco), 55 µM 2-mercaptoethanol (Gibco), 100 U/ml penicillin (Gibco), 100 µg/ml streptomycin (Gibco), and 10% fetal bovine serum (Gibco)). Cultures were supplemented on day 4 with 20 U/ml rIL2 (Roche, Basel, Switzerland) and then every 3–4 days with fresh media^36^.

### Intracellular staining

Expanded A68/NP_145_^+^ CD8^+^ T cells were stimulated with 1 µM peptide (DATYQRTRALVR, DTTYQRTRALVR, DVTYQRTRALVR or pool of DATYQRTRALVR, DTTYQRTRALVR, and DVTYQRTRALVR) and cultured for 5 h in the presence of 10 U/ml rIL2 and Golgi Stop (BD Biosciences). Following activation, cells were surface stained for 30 min with human anti-CD3-BV510 (1:200, Biolegend #317332), anti-CD4-BV650 (1:200, BD Horizon #563875), anti-CD14-APC-H7 (1:100, BD Pharmingen #560180), anti-CD19-APC-H7 (1:100, BD Pharmingen #560177), anti-CD8-PerCPCy5.5 (1:100, BD Pharmingen #565310), Live/Dead near-infrared (1:800, Invitrogen), and PE-streptavidin-conjugated A68/NP_145_ (DATYQRTRALVR) tetramer. Cells were fixed with BD Fix-Perm buffer (BD Biosciences) for 20 min, before intracellular staining for 30 min with human anti-IFN-γ-V450 (1:100, BD Horizon #560371) in perm wash buffer (BD Biosciences). Cells were washed, acquired on the BD Fortessa (BD Biosciences) and analyzed using the Flowjo software (Treestar, OR, USA).

### Magnetic enrichment of NP_145_^+^CD8^+^ T cells ex vivo

PBMCs or T cell lines (1–5 × 10^7^) were thawed in cRPMI medium supplemented with 50 U/ml Benzonase (Novagen Merck). Cells were washed once in magnetic-activated cell sorting (MACS) buffer (PBS supplemented with 0.5% Bovine Serum Albumin (BSA) (Sigma) and 2 mM EDTA) and incubated with anti-human FcR block [20 µl/1 × 10^7^ cells] (Milteny Biotec) for 15 min on ice and subsequently stained with PE- and/or APC-streptavidin-conjugated tetramers [1:100 in MACS buffer] for 1 h at room temperature. After one wash, cells were incubated for 30 min on ice with 100 µl anti-PE and/or 100 µl anti-APC MicroBeads (Milteny Biotec) in 400 or 300 µl MACS buffer, respectively. Cells were washed twice before passing through a LS column (Milteny Biotec) to enrich for tetramer-positive cells^45,67^. Cells were then surfaced stained in MACS buffer using human anti-CD45RA-FITC (1:200, BD Biosciences #555488), anti-CD8-PerCP-Cy5.5 (1:200, BD Biosciences #565310), anti-CD27^−^APC (1:50, BD Biosciences #337169), or anti-CD27-BV711 (1:200, BD Horizon #563167), anti-CD3-AF700 (1:50, BD Biosciences #557943), anti-CD14-APC-Cy7 (1:100, BD Biosciences #560180), anti-CD19 (1:100, BD Biosciences #560177), anti-CD62L-V450 (1:100, eBioscience #48-0629-42), anti-CD4-BV650 (1:100), anti-CD56-BV785 (1:100, BD Horizon #564058), anti-CD95-PECF594 (1:100, BD Horizon #562395), anti-CCR7-PeCy7 (1:50, BD Biosciences #557648), and Live/Dead fixable aqua dead-cell stain (1:800, Invitrogen) for 30 min on ice. Cells were subsequently washed once and fixed with 1% paraformaldehyde (ProSciTec) for acquiring on a LSRFortesa II (BD Biosciences) or resuspended in MACS buffer for single cell-(index)-sorting using a BD FACSAria III (BD Biosciences), followed by the analysis using a FlowJo software (BD Biosciences).

### Single-cell RT-PCR and TCR sequencing

A68/NP_145_ enriched cells were individually (index-) sorted into chilled 96-well twin.tec PCR plates (Eppendorf, Hamburg, Germany) and immediately stored at −80 °C until required. Single-cell paired CDR3α and CDR3β regions were analyzed by multiplex nested RT-PCR and followed by sequencing of the CDRα and CDRβ products^43,47,68^. Briefly, cDNA was synthesized from single cells in PCR plates in 2.5 µl reaction mixes, each containing 0.5 µl 5× VILO reaction mix (Invitrogen), 0.25 µl 10× SuperScript enzyme mix (Invitrogen), and 0.1% Triton X-100 (Sigma), which were incubated at 25°C for 10 min, 42°C for 120 min, and 85 °C for 5 min. TCR transcripts from each cell were amplified by multiplex nested PCR in 25 µl reaction mixes containing 2.5 µl cDNA. First-round PCR was performed with 2.5 µl 10× PCR buffer (containing 15 mM MgCL2) (Qiagen), 0.5 µl of 10 mM dNTP (Invitrogen), 0.15 µl *Taq* DNA polymerase (5 units/µl) (Qiagen), 2.5 pmol each of the external sense TRAV and TRBV and external antisense TRAC and TRBC primers (Supplementary Table 4). A total of 2.5 µl aliquots of the first-round PCR products served as templates for two separate second-round PCRs that incorporated, respectively, an internal sense TRAV and internal sense TRAC primers (Supplementary Table 4) or internal sense TRBV and internal antisense TRBC primers (Supplementary Table 4). The second-round PCR used CoralLoad PCR buffer 10× (Qiagen) instead of 10× PCR buffer. PCR conditions for external and internal PCR round were 95 °C for 2 min, followed by 35 cycles of 95 °C for 20 s, 52°C for 20 s, and 72°C for 45 s, followed by one step of 72°C for 7 min. PCR products were purified and sequenced with the respective internal TRAC and TRBC primer (Supplementary Table 4). Sequences were analyzed with FlinchTV. V–J regions were identified by IMGT query (www.imgt.org/IMGT_vquest). TCR sequences were parsed using the TCRdist analytical pipeline^69^. Clonotypes were defined as single-cell TCRαβ pairs that exhibit the same V, J, and CDR3 regions. Circos plots were generated using the online Circos software package (http://mkweb.bcgsc.ca)^70^.

### Statistical analysis

The data were analyzed by SPSS statistics 25 using a Mann–Withney test and differences were considered significant at a *p* value of <0.05. Medium and high responder groups were pooled to ensure adequate power for statistical analysis.

### Reporting summary

Further information on research design is available in the Nature Research Reporting Summary linked to this article.

## Supplementary information

Supplementary Information

Reporting Summary

## Data Availability

PDB accession number for the HLA−A*68:01-NP_145_ complex structure is 6PBH. TCR sequence data have been deposited into VDJdb [https://vdjdb.cdr3.net]. The source data underlying Figs. 2–8, Supplementary Figs. 2–4, Table 2 and Supplementary Table 3 are provided as a Source Data file. All other data are available from the authors upon request.

## Peer Review File

Peer Review File

## Source Data

Source Data
