# Supplementary material for: Challenging immunodominance of influenza-specific CD8+ T cell responses restricted by the risk-associated HLA-A*68:01 allomorph
Source: Nat Commun. 2019 Dec 6;10:5579. doi: 10.1038/s41467-019-13346-4 (PMC6898063; doi:10.1038/s41467-019-13346-4)
Supplement: Supplementary file 1 — Supplementary Information [file 41467_2019_13346_MOESM1_ESM.pdf]

# Supplementary Information

## Challenging immunodominance of influenza-specific CD8$^{+}$ T cell responses restricted by the risk-associated HLA-A*68:01 allomorph

van de Sandt CE, Clemens EB et al.

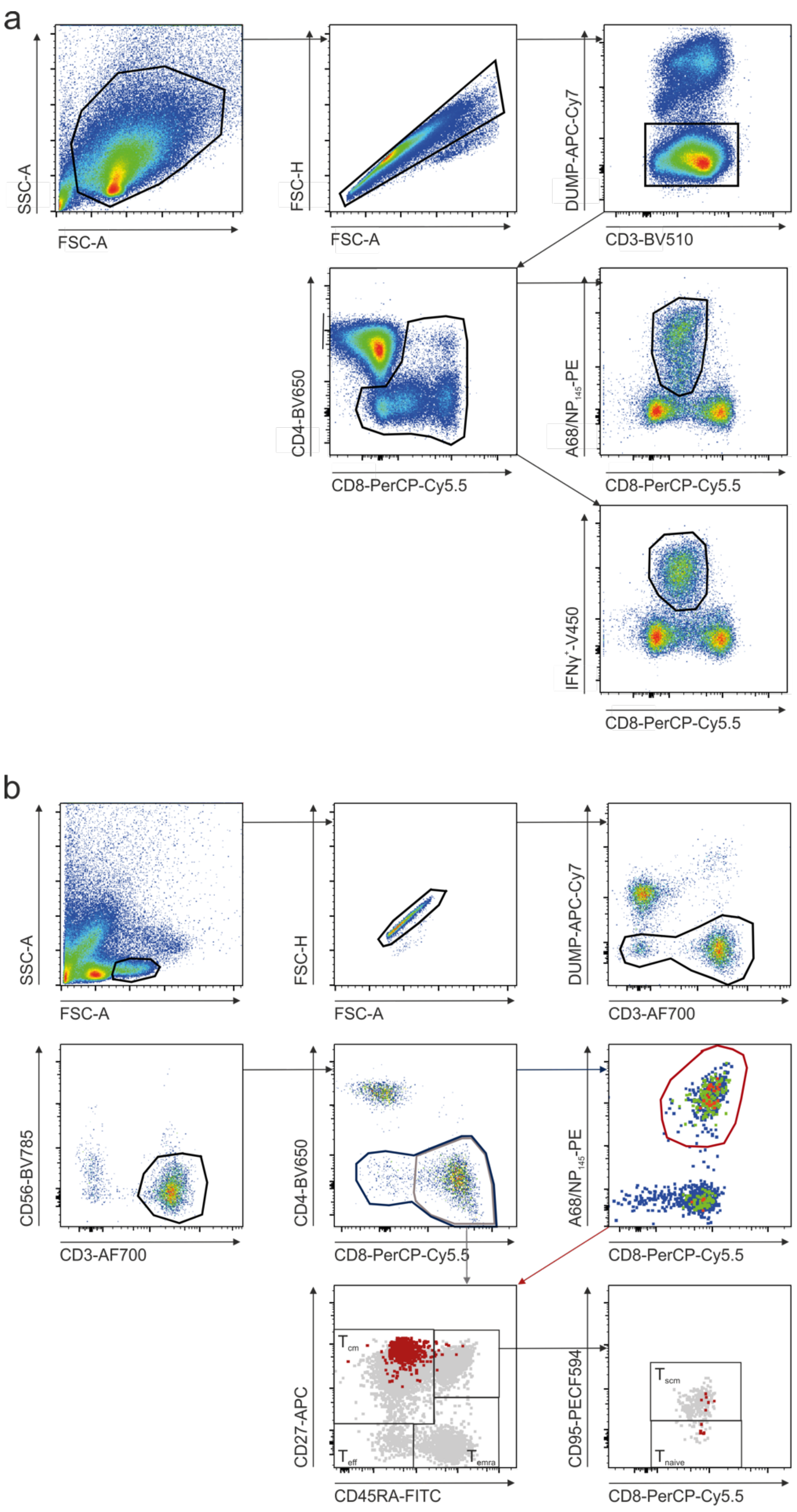


**Supplementary Figure 1. Gating strategy for identification of A68/NP$_{145}$$^{+}$CD8$^{+}$ T cells.** Representative FACS panels for gating of **(a)** A68/NP$_{145}$$^{+}$ and IFNγ-producing CD8$^{+}$ T cells presented on Figure 2b,c; and **(b)** naïve-like and memory phenotypes within CD8$^{+}$ T cells (depicted in grey) and A68/NP$_{145}$$^{+}$CD8$^{+}$ T cells (depicted in red) presented on Figure 3 and 4. Red gate indicates the sorting gate used for Figure 5, 6, 7 and 8.

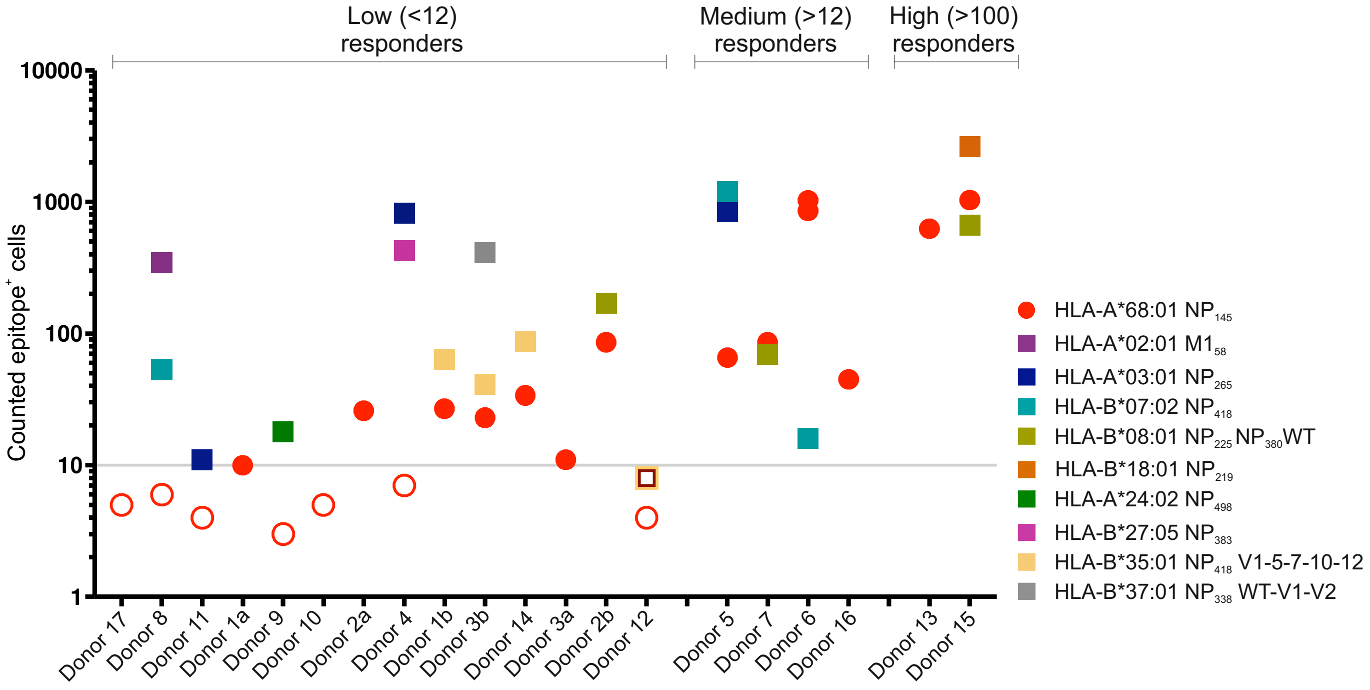


**Supplementary Figure 2. Total counts of A68/NP$_{145}$$^{+}$ CD8$^{+}$ T cells.**

Total number of A68/NP$_{145}$- and universal influenza peptide-specific CD8$^{+}$ T cells counted in the enriched sample by FACS analyzes is shown (n=17 donors). Detection limit for defining phenotypes was set at 10 counted cells.

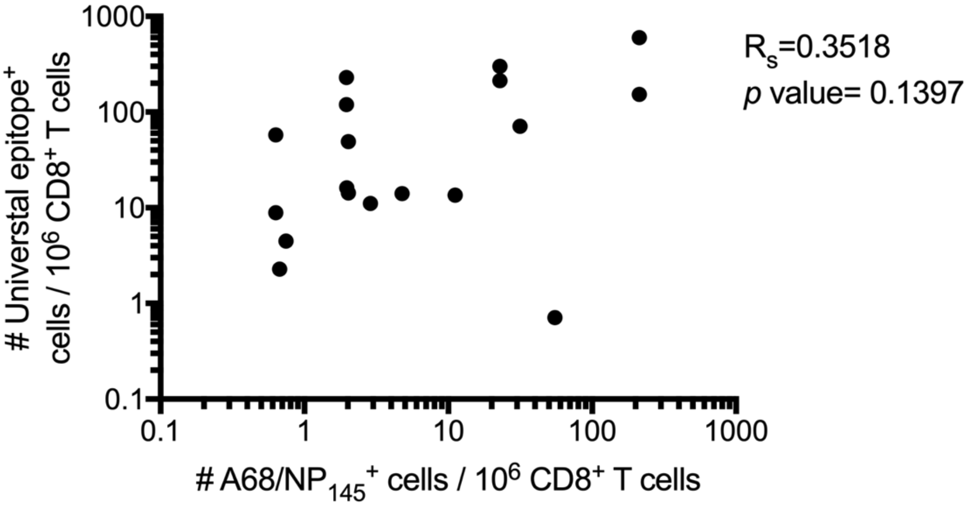


**Supplementary Figure 3. A68/$NP_{145}^{+}$ and universal $CD8^{+}$ T cell frequencies.**

A correlation analysis between the frequency of A68/$NP_{145}$-specific $CD8^{+}$ T cells and universal epitope-specific $CD8^{+}$ T cells (n=13) was performed using Spearman's correlation coefficient ($r_s$).

| Donor 16 High MFI | | | | | | | | |
|---|---|---|---|---|---|---|---|---|
| Clone ID | TRBV | TRBJ | CDR3b | CDR3b Length | TRAV | TRAJ | CDR3a | CDR3a Length |
| S1 | TRBV28 | TRBJ1-1 | CASSLIQANTEAFF | 10 | TRAV12-1 | TRAJ42 | CVVNVGYGGSQGNLIF | 11 |
| S2 | TRBV6-1 | TRBJ1-5 | CASSGNGQDLQPQHF | 10 | TRAV12-1 | TRAJ11 | CVVNRVENSGYSTLTF | 11 |
| S3 | TRBV27 | TRBJ2-7 | CASNPVIGDEQYF | 8 | TRAV4 | TRAJ40 | CLVGDHSGTYKYIF | 9 |
| S4 | TRBV28 | TRBJ1-1 | CASSLIQANTEAFF | 10 | - | - | - | - |
| S5 | TRBV12-3 | TRBJ1-1 | CASSTGTGVFEDTEAFF | 12 | TRAV4 | TRAJ6 | CLVGDGAGGSYIPTF | 10 |
| S6 | TRBV7-2 | TRBJ2-5 | CASSSIGVAGEETQYF | 11 | TRAV12-1 | TRAJ11 | CVVNVLLNSGYSTLTF | 11 |
| S7 | TRBV2 | TRBJ2-1 | CASNDPPGATNNEQFF | 11 | TRAV4 | TRAJ42 | CLVGGGSQGNLIF | 8 |
| S8 | TRBV9 | TRBJ1-6 | CASSESPIGDEPLHF | 10 | TRAV12-1 | TRAJ20 | CVVNPPPDDYKLSF | 9 |
| S9 | TRBV27 | TRBJ2-7 | CASNPVIGDEQYF | 8 | - | - | - | - |
| S10 | TRBV20-1 | TRBJ2-7 | CSGSQDPYEQYF | 7 | TRAV12-2 | TRAJ43 | CAVNNNNDMRF | 6 |
| S11 | TRBV5-4 | TRBJ2-7 | CASSWTQGDEQYF | 8 | TRAV4 | TRAJ40 | CLVGDHSGTYKYIF | 9 |
| S12 | TRBV20-1 | TRBJ1-4 | CSARDRGREKLFF | 8 | TRAV4 | TRAJ13 | CLVGDLPSGGYQKVTF | 11 |
| S13 | TRBV5-4 | TRBJ2-7 | CASSWTQGDEQYF | 8 | TRAV4 | TRAJ9 | CLVGDMAGGFKTIF | 9 |
| S14 | TRBV9 | TRBJ2-3 | CASSVELSTDTQYF | 9 | TRAV4 | TRAJ42 | CLVGGGSQGNLIF | 8 |
| S15 | TRBV28 | TRBJ1-1 | CASSLIQANTEAFF | 10 | TRAV12-1 | TRAJ11 | CVVNRVENSGYSTLTF | 11 |
| S16 | - | - | - | - | TRAV12-1 | TRAJ42 | CVVNVGYGGSQGNLIF | 11 |
| S17 | TRBV6-1 | TRBJ1-5 | CASSGNGQDLQPQHF | 10 | TRAV12-1 | TRAJ42 | CVVNVGYGGSQGNLIF | 11 |
| S18 | - | - | - | - | TRAV12-1 | TRAJ11 | CVVNVLLNSGYSTLTF | 11 |

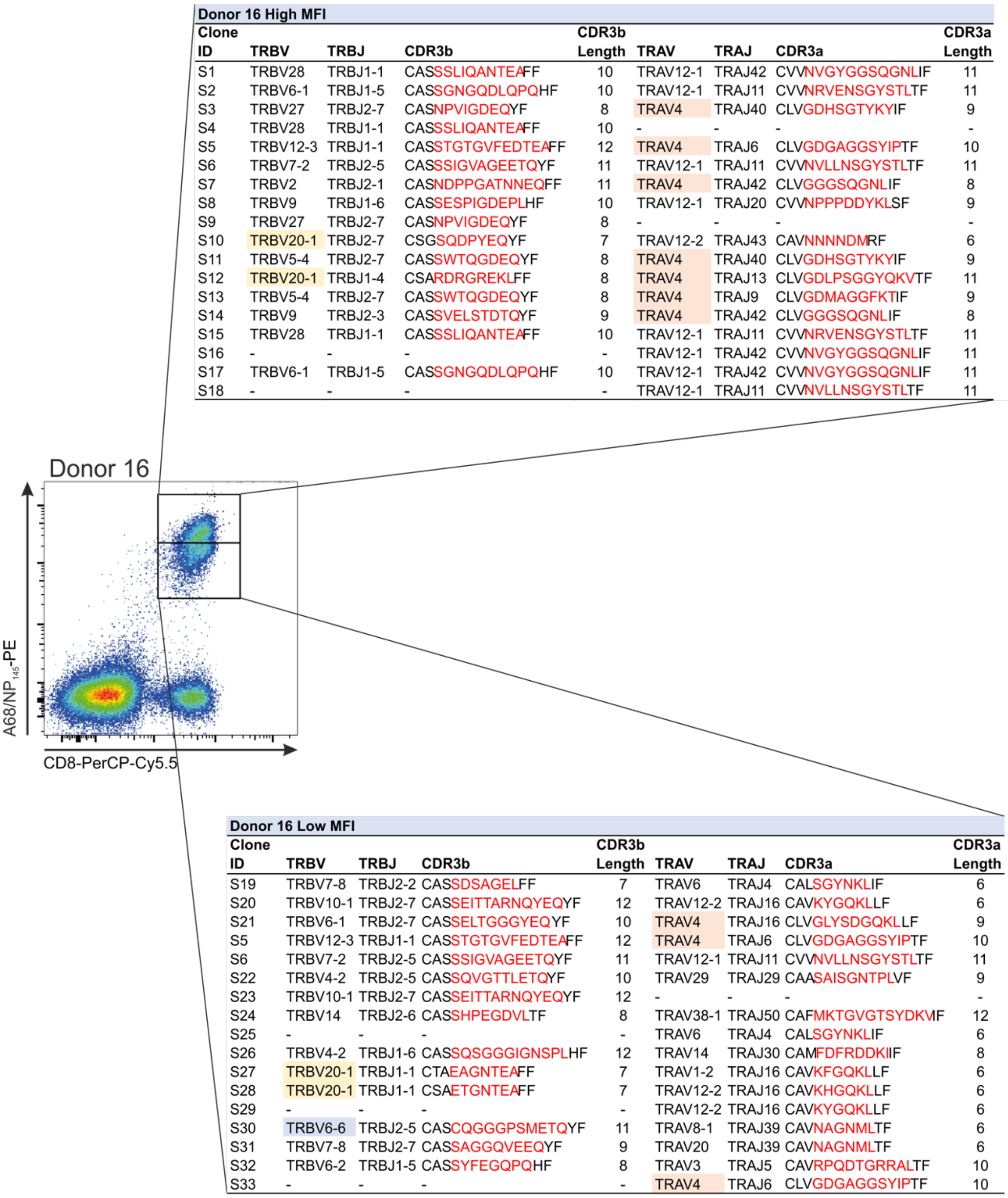


| Donor 16 Low MFI | | | | | | | | |
|---|---|---|---|---|---|---|---|---|
| Clone ID | TRBV | TRBJ | CDR3b | CDR3b Length | TRAV | TRAJ | CDR3a | CDR3a Length |
| S19 | TRBV7-8 | TRBJ2-2 | CASSDSAGELFF | 7 | TRAV6 | TRAJ4 | CALSGYNKLIF | 6 |
| S20 | TRBV10-1 | TRBJ2-7 | CASSEITTARNQYEQYF | 12 | TRAV12-2 | TRAJ16 | CAVKYGQKLLF | 6 |
| S21 | TRBV6-1 | TRBJ2-7 | CASSELTGGGYEQYF | 10 | TRAV4 | TRAJ16 | CLVGLYSDGQKLLF | 9 |
| S5 | TRBV12-3 | TRBJ1-1 | CASSTGTGVFEDTEAFF | 12 | TRAV4 | TRAJ6 | CLVGDGAGGSYIPTF | 10 |
| S6 | TRBV7-2 | TRBJ2-5 | CASSSIGVAGEETQYF | 11 | TRAV12-1 | TRAJ11 | CVVNVLLNSGYSTLTF | 11 |
| S22 | TRBV4-2 | TRBJ2-5 | CASSQVGTTLETQYF | 10 | TRAV29 | TRAJ29 | CAASAISGNTPLVF | 9 |
| S23 | TRBV10-1 | TRBJ2-7 | CASSEITTARNQYEQYF | 12 | - | - | - | - |
| S24 | TRBV14 | TRBJ2-6 | CASSHPEGDVLTF | 8 | TRAV38-1 | TRAJ50 | CAFMKTGVGTSYDKVIF | 12 |
| S25 | - | - | - | - | TRAV6 | TRAJ4 | CALSGYNKLIF | 6 |
| S26 | TRBV4-2 | TRBJ1-6 | CASSQSGGGIGNSPLHF | 12 | TRAV14 | TRAJ30 | CAMFDFRDDKIIF | 8 |
| S27 | TRBV20-1 | TRBJ1-1 | CTAEAGNTEAFF | 7 | TRAV1-2 | TRAJ16 | CAVKFGQKLLF | 6 |
| S28 | TRBV20-1 | TRBJ1-1 | CSAETGNTEAFF | 7 | TRAV12-2 | TRAJ16 | CAVKHGQKLLF | 6 |
| S29 | - | - | - | - | TRAV12-2 | TRAJ16 | CAVKYGQKLLF | 6 |
| S30 | TRBV6-6 | TRBJ2-5 | CASCQGGGPSMETQYF | 11 | TRAV8-1 | TRAJ39 | CAVNAGNMLTF | 6 |
| S31 | TRBV7-8 | TRBJ2-7 | CASSAGGQVEEQYF | 9 | TRAV20 | TRAJ39 | CAVNAGNMLTF | 6 |
| S32 | TRBV6-2 | TRBJ1-5 | CASSYFEGQPQHF | 8 | TRAV3 | TRAJ5 | CAVRPQDTGRRALTF | 10 |
| S33 | - | - | - | - | TRAV4 | TRAJ6 | CLVGDGAGGSYIPTF | 10 |

**Supplementary Figure 4. TCRαβ clonotype distribution based on A68/$NP_{145}$- avidity.** FACS panel shows gating of high and low avidity A68/$NP_{145}^{+}CD8^{+}$ T cells of a medium responder (donor 16). Clonotypes are arranged based on high and low A68/$NP_{145}$–tetramer avidity gates. This donor was sorted based on the low and high avidity gate before index-sorting was available, hence individual clonotypes could not be retraced to their exact tetramer-specific MFI. TRBV20-1 (yellow) and TRAV4 (orange) are commonly observed clonotypes which are shared between donors.

**Supplementary Table 1 Data collection and refinement statistics (molecular replacement)**

| | HLA-A*68:01-NP145-156 |
|---|---|
| **Data collection** | |
| Space group | $P2_12_12_1$ |
| Cell dimensions | |
| *a*, *b*, *c* (Å) | 59.75, 79.00, 112.65 |
| α, β, γ (°) | 90.00, 90.00, 90.00 |
| Resolution (Å) | 47.66 – 1.89 (2.00 – 1.89) |
| $R_{pim}$ | 3.9 (26.9) |
| $I / \sigma I$ | 13.3 (3.3) |
| Completeness (%) | 99.9 (99.4) |
| Redundancy | 7.2 (7.3) |
| | |
| **Refinement** | |
| Resolution (Å) | 27.67 – 1.89 |
| No. reflections | 43204 |
| $R_{work}$ / $R_{free}$ | 0.195/0.233 |
| No. atoms | |
| Protein | 3098 |
| Ligand/ion | 14 |
| Water | 347 |
| *B*-factors (Å$^2$) | |
| Protein | 27.7 |
| Ligand/ion | 64.0 |
| Water | 39.7 |
| R.m.s. deviations | |
| Bond lengths (Å) | 0.010 |
| Bond angles (°) | 1.02 |

Data collected from single crystal. *Values in parentheses are for highest-resolution shell.

[AU: Equations defining various *R*-values are standard and hence are no longer defined in the footnotes.]
[AU: Ramachandran statistics should be in Methods section at the end of Refinement subsection.]
[AU: Wavelength of data collection, temperature and beamline should all be in Methods section.]

## Supplementary Table 2. Frequency amino acid variations in $NP_{145-156}$ peptide

| | | | | Position $NP_{145-156}$ peptide | | | | | | | | | | | | | | | | | | | | | | | | | | | | | | | | | | | |
|---|---|---|---|---|---|---|---|---|---|---|---|---|---|---|---|---|---|---|---|---|---|---|---|---|---|---|---|---|---|---|---|---|---|---|---|---|---|---|---|
| Virus[*] | | | | 145 | | | 146[§] | | | 147 | | | 148 | | | 149 | | | 150 | | | 151 | | | 152 | | | 153 | | | 154 | | | 155 | | | 156 | | |
| Species | Serotype | Year | Total | aa | % | # | aa | % | # | aa | % | # | aa | % | # | aa | % | # | aa | % | # | aa | % | # | aa | % | # | aa | % | # | aa | % | # | aa | % | # | aa | % | # |
| Human | H1N1 | 1918-<br>1957 | 77 | D | 100 | 77 | T†<br>A | 53.2<br>46.8 | 41<br>36 | T | 100 | 77 | Y | 100 | 77 | Q | 100 | 77 | R | 100 | 77 | T | 100 | 77 | R | 100 | 77 | A | 100 | 77 | L | 100 | 77 | V | 100 | 77 | R | 100 | 77 |
| Human | H2N2 | 1957-<br>1968 | 119 | D | 100 | 119 | T | 100 | 119 | T | 100 | 119 | Y | 100 | 119 | Q | 100 | 119 | R | 100 | 119 | T | 100 | 119 | R | 100 | 119 | A | 100 | 119 | L | 100 | 119 | V | 100 | 119 | R | 100 | 119 |
| Human | H3N2 | 1968-<br>2018 | 13497 | D | 100 | 13497 | A¶<br>T<br>V | 91.8<br>8.16<br>0.05 | 12388<br>1102<br>7 | T<br>I | 99.9<br>0.01 | 13495<br>2 | Y | 100 | 13497 | Q<br>H | 99.9<br>0.01 | 13496<br>1 | R | 100 | 13497 | T | 100 | 13497 | R<br>K | 99.9<br>0.01 | 13496<br>1 | A | 100 | 13497 | L<br>I | 99.9<br>0.04 | 13492<br>5 | V | 100 | 13497 | R | 100 | 13498 |
| Human | H1N1 | 1977-<br>2008 | 1132 | D | 100 | 1132 | T<br>A | 98.9<br>1.1 | 1119<br>13 | T | 100 | 1132 | Y | 100 | 1132 | Q | 100 | 1132 | R | 100 | 1132 | T | 100 | 1132 | R | 100 | 1132 | A | 100 | 1132 | L | 100 | 1132 | V | 100 | 1132 | R | 100 | 1132 |
| Human | H1N1 | 2009-<br>2018 | 9291 | D<br>G | 99.9<br>0.01 | 9290<br>1 | A°<br>T | 97<br>3 | 9012<br>279 | T | 100 | 9291 | Y | 100 | 9291 | Q<br>H | 99.9<br>0.01 | 9290<br>1 | R | 100 | 9291 | T<br>A | 99.9<br>0.01 | 9290<br>1 | R | 100 | 9291 | A<br>V | 99.9<br>0.01 | 9290<br>1 | L<br>I | 99.9<br>0.01 | 9290<br>1 | V | 100 | 9291 | R<br>L<br>C | 99.9<br>0.03<br>0.02 | 9286<br>3<br>2 |
| Human | H5N1 | 1997-<br>2014 | 194 | D | 100 | 194 | A<br>T | 99.5<br>0.5 | 193<br>1 | R | 100 | 194 | Y | 100 | 194 | Q | 100 | 194 | R | 100 | 194 | T | 100 | 194 | R | 100 | 194 | A | 100 | 194 | L | 100 | 194 | V | 100 | 194 | R | 100 | 194 |
| Human | H7N9 | 2013-<br>2017 | 98 | D | 100 | 98 | A | 100 | 98 | T | 100 | 98 | Y | 100 | 98 | Q | 100 | 98 | R | 100 | 98 | T | 100 | 98 | R | 100 | 98 | A | 100 | 98 | L | 100 | 98 | V | 100 | 98 | R | 100 | 98 |

* Amino acid sequences present in the National Center for Biotechnology Information (NCBI; http:/www.ncbi.nlm.nih.gov/genomes/FLU) database at July 31st 2018
Year = year of isolation according to database; aa = amino acid; % = frequency; # = absolute number of viruses
§ unless otherwise indicated we used peptide variant with an “A” at position 146 in this study
† Amino acid at position 146 gradually changes from “A” to “T” in time, T is fixed from 1949 onwards
¶ Amino acid at position 146 gradually changes from “T” to “A” in time, A is fixed from 2001 onwards
° All viruses with “T” at position 146 are from 2009 and come in clusters

## Supplementary Table 3. Paired TRBV-TRBJ/TRAV-TRAJ clonotype frequencies of A68/NP$_{145}^{+}$-specific TCRs

| | | | | | | | | | Low (<12) responders | | | | Medium (>20) responders | | | | High (>100) responder |
|---|---|---|---|---|---|---|---|---|---|---|---|---|---|---|---|---|---|
| **Clone ID** | **TRBV** | **TRBJ** | **CDR3b** | **CDR3b length** | **TRAV** | **TRAJ** | **CDR3a** | **CDR3a length** | **Donor 1b** | **Donor 3b** | **Donor 3a** | **Donor 2b** | **Donor 5*** | **Donor 7** | **Donor 6** | **Donor 16*** | **Donor 13*** |
| A | TRBV4-2 | TRBJ2-7 | CASSQAGSSYEQYF | 9 | - | - | - | - | 1 | | | | | | | | |
| B | TRBV11-2 | TRBJ2-5 | CASSLDLPGPEGETQYF | 12 | TRAV17 | TRAJ22 | CATDAARQLTF | 6 | 1 | | | | | | | | |
| C | TRBV20-1 | TRBJ1-1 | CSAEQGNTEAFF | 7 | TRAV12-2 | TRAJ49 | CAVKVSNQFYF | 6 | 1 | | | | | | | | |
| D | - | - | - | - | TRAV20 | TRAJ15 | CAVQANQAGTALIF | 9 | 1 | | | | | | | | |
| E | TRBV2 | TRBJ2-7 | CASSPGTGGNEQYF | 9 | TRAV38-1 | TRAJ52 | CAYRSAGGGTSYGKLTF | 12 | 1 | | | | | | | | |
| F | TRBV2 | TRBJ1-1 | CASSEVGVFEAFF | 8 | TRAV13-2 | TRAJ34 | CAEKRPTDKLIF | 7 | | 1 | | | | | | | |
| G | - | - | - | - | TRAV14 | TRAJ42 | CAMSIFYGGSQGNLIF | 11 | | 1 | | | | | | | |
| H | TRBV14 | TRBJ1-1 | CASSLGVNTEAFF | 8 | TRAV38-1 | TRAJ43 | CAYRSSFDMRF | 6 | | 1 | | | | | | | |
| I | - | - | - | - | TRAV4 | TRAJ17 | CLVGDIGAAGNKLTF | 10 | | 1 | | | | | | | |
| J | TRBV2 | TRBJ1-1 | CASRVVEGIAEAFF | 9 | - | - | - | - | | | 1 | | | | | | |
| K | TRBV6-1 | TRBJ2-7 | CASSAGLAPDEQYF | 9 | - | - | - | - | | | 1 | | | | | | |
| L | TRBV13 | TRBJ2-3 | CASSSPAGGPTDTQYF | 11 | - | - | - | - | | | 1 | | | | | | |
| M | TRBV6-1 | TRBJ1-2 | CASNEDSNYGYTF | 8 | TRAV1-2 | TRAJ33 | CAAMDSNYQLIW | 7 | | | 1 | | | | | | |
| N | TRBV4-1 | TRBJ1-4 | CASSQVGPNEKLFF | 9 | TRAV29 | TRAJ41 | CAASDPNSGYALNF | 9 | | | 1 | | | | | | |
| O | TRBV27 | TRBJ1-2 | CASSLGGYGYTF | 7 | TRAV24 | TRAJ39 | CAFMGGAGNMLTF | 8 | | | 1 | | | | | | |
| P | TRBV4-1 | TRBJ2-5 | CASSQVGPGETQYF | 9 | TRAV35 | TRAJ34 | CAGYYNTDKLIF | 7 | | | 1 | | | | | | |
| Q | TRBV4-1 | TRBJ1-1 | CASSQDPWAGSAFF | 9 | TRAV19 | TRAJ15 | CALSYLGLQAGTALIF | 11 | | | 1 | | | | | | |
| R | - | - | - | - | TRAV12-3 | TRAJ23 | CASYNQGGKLIF | 7 | | | 1 | | | | | | |
| S | TRBV6-1 | TRBJ1-2 | CASSELEAYGYTF | 8 | TRAV8-3 | TRAJ33 | CAVGERDSNYQLIW | 9 | | | 1 | | | | | | |
| T | TRBV5-5 | TRBJ1-2 | CASSPGTDYGYTF | 8 | TRAV20 | TRAJ15 | CAVHPNQAGTALIF | 9 | | | 1 | | | | | | |
| U | TRBV6-4 | TRBJ2-3 | CASSAGTSPTDTQYF | 10 | TRAV1-2 | TRAJ12 | CAVMDSSYKLIF | 7 | | | 1 | | | | | | |
| V | TRBV6-1 | TRBJ2-7 | CASSDGDHSYEQYF | 9 | TRAV1-2 | TRAJ12 | CAVMDSSYKLIF | 7 | | | 1 | | | | | | |
| W | TRBV14 | TRBJ1-5 | CASSQGGPVGNQPQHF | 11 | TRAV8-1 | TRAJ23 | CAVNARNQGGKLIF | 9 | | | 1 | | | | | | |
| X | TRBV7-2 | TRBJ1-1 | CGGGYMGTEAFF | 7 | TRAV20 | TRAJ15 | CAVQANQAGTALIF | 9 | | | 1 | | | | | | |
| Y | TRBV7-9 | TRBJ1-2 | CASSFPSRGANYGYTF | 11 | TRAV20 | TRAJ53 | CAVRSEGGSNYKLTF | 10 | | | 1 | | | | | | |
| Z | TRBV24-1 | TRBJ2-3 | CATSDLGLAGDTQYF | 10 | TRAV38-1 | TRAJ45 | CAYRSPYSGGGADGLTF | 12 | | | 1 | | | | | | |
| AA | TRBV15 | TRBJ1-1 | CATSVTGDPNTEAFF | 10 | TRAV4 | TRAJ29 | CLIPGVSGNTPLVF | 9 | | | 1 | | | | | | |
| AB | TRBV5-4 | TRBJ2-5 | CASSLVPSDQETQYF | 10 | TRAV4 | TRAJ15 | CLVGDGKAGTALIF | 9 | | | 1 | | | | | | |
| AC | TRBV4-2 | TRBJ2-3 | CASSQDPDTQYF | 7 | TRAV4 | TRAJ43 | CLVGPFMRF | 4 | | | 1 | | | | | | |
| AD | TRBV6-1 | TRBJ2-7 | CASASGGFGDEQYF | 9 | TRAV4 | TRAJ22 | CLVVSARQLTF | 6 | | | 1 | | | | | | |
| AE | TRBV30 | TRBJ1-1 | CAWSVTSTGGTEAFF | 10 | TRAV12-1 | TRAJ8 | CVVNMIMNTGFQKLVF | 11 | | | 1 | | | | | | |
| AF | TRBV20-1 | TRBJ1-1 | CSAETGNTEAFF | 7 | - | - | - | - | | | | 2 | | | | | |

| | | | | | | | | | | | | |
|---|---|---|---|---|---|---|---|---|---|---|---|---|
| AG | TRBV4-3 | TRBJ2-1 | CANPPGSSYNEQFF | 9 | - | - | - | - | 1 | | | |
| AH | TRBV6-4 | TRBJ2-2 | CASRPGQHTGELFF | 9 | - | - | - | - | 1 | | | |
| AI | TRBV9 | TRBJ2-1 | CASSEVADYNEQFF | 9 | - | - | - | - | 1 | | | |
| AJ | TRBV5-4 | TRBJ1-1 | CASSLEGDTEAFF | 8 | TRAV20 | TRAJ15 | CAALANQAGTALIF | 9 | 1 | | | |
| AK | TRBV9 | TRBJ2-2 | CASSVGDLLTGELFF | 10 | TRAV13-2 | TRAJ10 | CAERIRKGQVLTGGGNKLTF | 15 | 1 | | | |
| AL | TRBV29-1 | TRBJ2-1 | CSFRDLSSYNEQFF | 9 | TRAV35 | TRAJ17 | CAGLKAAGNKLTF | 8 | 1 | | | |
| AM | TRBV2 | TRBJ2-1 | CASSEFQGDNEQFF | 9 | TRAV38-1 | TRAJ52 | CAHRSAGGGTSYGKLTF | 12 | 1 | | | |
| AN | - | - | - | - | TRAV27 | TRAJ43 | CAKYNNNDMRF | 6 | 1 | | | |
| AO | - | - | - | - | TRAV19 | TRAJ49 | CALSEAGTGNQFYF | 9 | 1 | | | |
| AP | TRBV25-1 | TRBJ2-1 | CASSDGSFNEQFF | 8 | TRAV3 | TRAJ31 | CAVDNARLMF | 5 | 1 | | | |
| AQ | TRBV20-1 | TRBJ2-3 | CSATRLAGGPTDTQYF | 11 | TRAV8-3 | TRAJ43 | CAVGPGSNNDMRF | 8 | 1 | | | |
| AR | - | - | - | - | TRAV1-2 | TRAJ45 | CAVTGGGADGLTF | 8 | 1 | | | |
| AS | TRBV9 | TRBJ2-1 | CASSEVADYNEQFF | 9 | TRAV26-2 | TRAJ41 | CILTHSNSGYALNF | 9 | 1 | | | |
| AT | TRBV19 | TRBJ2-5 | CASSVALGNQETQYF | 10 | TRAV4 | TRAJ11 | CLVGDGAGYSTLTF | 9 | 1 | | | |
| AU | - | - | - | - | TRAV4 | TRAJ6 | CLVGDGSGGSYIPTF | 10 | 1 | | | |
| AV | TRBV19 | TRBJ2-5 | CASSPALGDQETQYF | 10 | TRAV4 | TRAJ11 | CLVGDGSGYSTLTF | 9 | 1 | | | |
| AW | TRBV29-1 | TRBJ2-2 | CSVEEGLQTGELFF | 9 | TRAV4 | TRAJ8 | CLVGDLGNTGFQKLVF | 11 | 1 | | | |
| AX | TRBV24-1 | TRBJ2-5 | CATSDLEGWTQYF | 8 | TRAV12-1 | TRAJ42 | CVVTSAGSQGNLIF | 9 | 1 | | | |
| AY | TRBV20-1 | TRBJ2-1 | CSADNVAGGPGSEQFF | 11 | TRAV13-1 | TRAJ43 | CATYDMRF | 3 | | 3 | | |
| AZ | TRBV6-1 | TRBJ2-1 | CASSEPRDEQFF | 7 | TRAV1-2 | TRAJ9 | CAVETGGFKTIF | 7 | | 3 | | |
| BA | TRBV20-1 | TRBJ2-7 | CSASQDPYEQYF | 7 | TRAV35 | TRAJ37 | CAGQTTSNTGKLIF | 9 | | 2 | | |
| BB | TRBV20-1 | TRBJ2-1 | CSALYPLAGPGNEQFF | 11 | TRAV26-2 | TRAJ30 | CILNRDDKIIF | 6 | | 2 | | |
| BC | TRBV12-4 | TRBJ1-4 | CASSFLGPEGEKLFF | 10 | TRAV13-1 | TRAJ42 | CAASIRGSQGNLIF | 9 | | 1 | | |
| BD | TRBV4-2 | TRBJ2-1 | CASSQEGAGADEQFF | 10 | TRAV13-1 | TRAJ43 | CAASPRNNDMRF | 7 | | 1 | | |
| BE | TRBV12-4 | TRBJ1-4 | CASSLIFRDGEKLFF | 10 | TRAV17 | TRAJ42 | CATGPIYGGSQGNLIF | 11 | | 1 | | |
| BF | TRBV20-1 | TRBJ2-7 | CSASQDPYEQYF | 7 | TRAV17 | TRAJ42 | CATGPIYGGSQGNLIF | 11 | | 1 | | |
| BG | TRBV29-1 | TRBJ1-1 | CSGETGNTEAFF | 7 | TRAV12-2 | TRAJ3 | CAVKRSASKIIF | 7 | | 1 | | |
| BH | TRBV20-1 | TRBJ2-7 | CSASQDPYEQYF | 7 | TRAV12-2 | TRAJ43 | CAVNGNNDMRF | 6 | | 1 | | |
| BI | TRBV27 | TRBJ2-3 | CASSPGTPTDTQYF | 9 | TRAV1-2 | TRAJ16 | CAVSLDGQKLLF | 7 | | 1 | | |
| BJ | TRBV6-6 | TRBJ2-3 | CASSASRVGEDTQYF | 10 | TRAV1-1 | TRAJ37 | CAVSSNTGKLIF | 7 | | 1 | | |
| BK | TRBV20-1 | TRBJ2-1 | CSALYPLAGPGNEQFF | 11 | TRAV26-2 | TRAJ30 | CILNRDDKIIF | 6 | | 1 | | |
| BL | TRBV6-1 | TRBJ2-7 | CASSETTGPAYEQYF | 10 | TRAV4 | TRAJ16 | CLVGLFSDGQKLLF | 9 | | 1 | | |
| BM | TRBV6-6 | TRBJ2-1 | CASSSPSGVYNEQFF | 10 | TRAV4 | TRAJ4 | CLVGDLINSGGYNKLIF | 12 | | | 18 | |
| BN | TRBV6-6 | TRBJ2-1 | CASSSPSGVYNEQFF | 10 | - | - | - | - | | | 9 | |
| BO | TRBV7-8 | TRBJ2-7 | CASTPGGAGDEQYF | 9 | TRAV35 | TRAJ42 | CAGHHYGGSQGNLIF | 10 | | | 1 | |
| BP | TRBV19 | TRBJ2-2 | CASSSGGDERYTGELFF | 12 | TRAV21 | TRAJ11 | CAVSPTLTF | 4 | | | 1 | |
| BQ | TRBV4-3 | TRBJ2-5 | CASSQVASDQETQYF | 10 | TRAV4 | TRAJ6 | CLVGDGVGGSYIPTF | 10 | | | 1 | |
| BR | TRBV20-1 | TRBJ1-4 | CSARDRREEKLFF | 8 | TRAV4 | TRAJ13 | CLVGDIAAGGYQKVTF | 11 | | | 1 | |
| BS | - | - | - | - | TRAV4 | TRAJ4 | CLVGDLINSGGYNKLIF | 12 | | | 1 | |
| BT | TRBV30 | TRBJ2-7 | CAWSPAGLAMYEQYF | 10 | - | - | - | - | | | | 5 |

| | | | | | | | | | | |
|---|---|---|---|---|---|---|---|---|---|---|
| BU | TRBV20-1 | TRBJ1-1 | CSAESGNTEAFF | 7 | - | - | - | - | 4 | |
| BV | TRBV30 | TRBJ2-7 | CAWSPAGLAMYEQYF | 10 | TRAV12-1 | TRAJ29 | CVVNANSGNTPLVF | 9 | 3 | |
| BW | TRBV6-1 | TRBJ2-7 | CASSEAGGPGYEQYF | 10 | - | - | - | - | 2 | |
| BX | TRBV29-1 | TRBJ1-4 | CSVRDISTNEKLFF | 9 | - | - | - | - | 2 | |
| BY | TRBV20-1 | TRBJ1-6 | CSAEDGNSPLHF | 7 | TRAV12-2 | TRAJ47 | CAVKYGNKLVF | 6 | 2 | |
| BZ | TRBV15 | TRBJ2-5 | CATSSEATGVGETQYF | 11 | - | - | - | - | 1 | |
| CA | TRBV20-1 | TRBJ1-1 | CSAEDGNTEAFF | 7 | - | - | - | - | 1 | |
| CB | TRBV20-1 | TRBJ1-4 | CSARDRVEEKLFF | 8 | - | - | - | - | 1 | |
| CC | TRBV4-3 | TRBJ2-1 | CASSQEPSGMVSRDNEQFF | 14 | TRAV1-2 | TRAJ9 | CAVETGGFKTIF | 7 | 1 | |
| CD | TRBV15 | TRBJ2-5 | CATSSEATGVGETQYF | 11 | TRAV21 | TRAJ47 | CAVGYGNKLVF | 6 | 1 | |
| CE | TRBV20-1 | TRBJ1-1 | CSAESGNTEAFF | 7 | TRAV12-2 | TRAJ26 | CAVKKGQNFVF | 6 | 1 | |
| CF | - | - | - | - | TRAV4 | TRAJ4 | CLVDPLAAGGYNKLIF | 11 | 1 | |
| CG | TRBV27 | TRBJ2-2 | CASSSRVGDTGELFF | 10 | TRAV4 | TRAJ4 | CLVDPLAAGGYNKLIF | 11 | 1 | |
| CH | - | - | - | - | TRAV4 | TRAJ42 | CLVGAGGSQGNLIF | 9 | 1 | |
| CI | TRBV6-6 | TRBJ2-1 | CASSSTAADEQFF | 8 | TRAV4 | TRAJ11 | CLVGDGSGYSTLTF | 9 | 1 | |
| CJ | TRBV28 | TRBJ2-2 | CASSTGTGGPSGTGELFF | 13 | TRAV12-1 | TRAJ24 | CVVNTDSWGKLQF | 8 | 1 | |
| CK | TRBV7-8 | TRBJ2-2 | CASSDSAGELFF | 7 | TRAV6 | TRAJ4 | CALSGYNKLIF | 6 | | 5 |
| CL | TRBV7-2 | TRBJ2-5 | CASSSIGVAGEETQYF | 11 | TRAV12-1 | TRAJ11 | CVVNVLLNSGYSTLTF | 11 | | 5 |
| CM | TRBV2 | TRBJ2-1 | CASNDPPGATNNEQFF | 11 | TRAV4 | TRAJ42 | CLVGGGSQGNLIF | 8 | | 4 |
| CN | TRBV28 | TRBJ1-1 | CASSSLIQANTEAFF | 10 | TRAV12-1 | TRAJ42 | CVVNVGYGGSQGNLIF | 11 | | 4 |
| CO | TRBV7-2 | TRBJ2-7 | CASSDLAGTSGTNTYEQYF | 14 | TRAV4 | TRAJ21 | CLVGGNFNKFYF | 7 | | 3 |
| CP | - | - | - | - | TRAV12-1 | TRAJ11 | CVVNRVENSGYSTLTF | 11 | | 3 |
| CQ | TRBV7-8 | TRBJ2-2 | CASSDSAGELFF | 7 | TRAV8-1 | TRAJ6 | CAANSGGSYIPTF | 8 | | 2 |
| CR | TRBV4-2 | TRBJ2-5 | CASSQVGTTLETQYF | 10 | TRAV29 | TRAJ29 | CAASAISGNTPLVF | 9 | | 2 |
| CS | TRBV9 | TRBJ2-6 | CASSVNPAQGSGANVLTF | 13 | TRAV9-2 | TRAJ34 | CALIYNTDKLIF | 7 | | 2 |
| CT | TRBV4-2 | TRBJ2-7 | CASSQSPTGAIHEQYF | 11 | - | - | - | - | | 1 |
| CU | TRBV27 | TRBJ1-5 | CASSTAGHQPQHF | 8 | - | - | - | - | | 1 |
| CV | TRBV7-8 | TRBJ2-7 | CASSLAGGLAGPSYEQYF | 13 | TRAV5 | TRAJ33 | CAEILMDSNYQLIW | 9 | | 1 |
| CW | TRBV4-3 | TRBJ2-2 | CASSQGGRTGELFF | 9 | TRAV13-2 | TRAJ25 | CAENKGQGFSFIF | 8 | | 1 |
| CX | TRBV9 | TRBJ2-3 | CASSVELSTDTQYF | 9 | TRAV19 | TRAJ44 | CALKGNTGTASKLTF | 10 | | 1 |
| CY | TRBV20-1 | TRBJ1-1 | CSAETGNTEAFF | 7 | TRAV1-2 | TRAJ16 | CAPKDGQKLLF | 6 | | 1 |
| CZ | TRBV20-1 | TRBJ2-1 | CSAETGNTEQFF | 7 | TRAV12-2 | TRAJ26 | CAVKWSGQNFVF | 7 | | 1 |
| DA | TRBV10-1 | TRBJ2-7 | CASSEITTARNQYEQYF | 12 | TRAV12-2 | TRAJ16 | CAVKYGGQKLLF | 6 | | 1 |
| DB | TRBV4-2 | TRBJ2-7 | CASSQSPTGAIHEQYF | 11 | TRAV4 | TRAJ40 | CLVGDASGTYKYIF | 9 | | 1 |
| DC | - | - | - | - | TRAV4 | TRAJ11 | CLVGDGSGYSTLTF | 9 | | 1 |
| DD | TRBV14 | TRBJ2-5 | CASSQVASNQETQYF | 10 | TRAV4 | TRAJ11 | CLVGDGSGYSTLTF | 9 | | 1 |
| DE | TRBV27 | TRBJ2-7 | CASNPVIGDEQYF | 8 | TRAV4 | TRAJ40 | CLVGDHSGTYKYIF | 9 | | 1 |
| DF | TRBV5-4 | TRBJ2-7 | CASSTPGDEQYF | 7 | TRAV4 | TRAJ9 | CLVGDHTGGFKTIF | 9 | | 1 |
| DG | TRBV20-1 | TRBJ1-4 | CSARDRGDEKLFF | 8 | TRAV4 | TRAJ13 | CLVGDMPSGGYQKVTF | 11 | | 1 |
| DH | TRBV20-1 | TRBJ1-4 | CSARDRGDEKLFF | 8 | TRAV4 | TRAJ13 | CLVGDVPSGGYQKVTF | 11 | | 1 |

| | | | | | | | | | | | |
|---|---|---|---|---|---|---|---|---|---|---|---|
| DI | TRBV9 | TRBJ1-6 | CASSESPIGDEPLHF | 10 | TRAV12-1 | TRAJ20 | CVVNPPPDDYKLSF | 9 | | 1 | |
| DJ | TRBV6-1 | TRBJ1-5 | CASSGNEQDLQPQHF | 10 | TRAV12-1 | TRAJ11 | CVVNRVENSGYSTLTF | 11 | | 1 | |
| DK | TRBV9 | TRBJ2-1 | CASSVDLKAGEEGEQFF | 12 | TRAV19 | TRAJ9 | CALSNTGGFKTIF | 8 | | | 18 |
| DL | TRBV3-1 | TRBJ1-2 | CASSHGGPPTFYGYTF | 11 | - | - | - | - | | | 1 |

* TCR based on T cell lines

CDR3 length was calculated based on red CDR3 sequence

**Supplementary Table 4. TCR sequencing primers**

| Primer Name | Sequence |
|---|---|
| huTRAV1ext | AACTGCACGTACCAGACATC |
| huTRAV2ext | GATGTGCACCAAGACTCC |
| huTRAV3ext | AAGATCAGGTCAACGTTGC |
| huTRAV4ext | CTCCATGGACTCATATGAAGG |
| huTRAV5ext | CTTTTCCTGAGTGTCCGAG |
| huTRAV6ext | CACCCTGACCTGCAACTATAC |
| huTRAV7ext | GCAAAATACAGGGATGGG |
| huTRAV8-1ext | CTCACTGGAGTTGGGATG |
| huTRAV8-3ext | CACTGTCTCTGAAGGAGCC |
| huTRAV8-2,4ext | GCCACCCTGGTTAAAGG |
| huTRAV8-6ext | GAGCTGAGGTGCAACTACTC |
| huTRAV8-7ext | CTAACAGAGGCCACCCAG |
| huTRAV9-1_2ext | TGGTATGTCCAATATCCTGG |
| huTRAV10ext | CAAGTGGAGCAGAGTCCTC |
| huTRAV12-1_3ext | CARTGTTCCAGAGGGAGC |
| huTRAV13-1ext | CATCCTTCAACCCTGAGTG |
| huTRAV13-2ext | CAGCGCCTCAGACTACTTC |
| huTRAV14ext | AAGATAACTCAAACCCAACCAG |
| huTRAV16ext | AGTGGAGCTGAAGTGCAAC |
| huTRAV17ext | GGAGAAGAGGATCCTCAGG |
| huTRAV18ext | TCCAGTATCTAAACAAAGAGCC |
| huTRAV19ext | AGGTAACTCAAGCGCAGAC |
| huTRAV20ext | CACAGTCAGCGGTTTAAGAG |
| huTRAV21ext | TTCCTGCAGCTCTGAGTG |
| huTRAV22ext | GTCCTCCAGACCTGATTCTC |
| huTRAV23ext | TGCTTATGAGAACACTGCG |
| huTRAV24ext | CTCAGTCACTGCATGTTCAG |
| huTRAV25ext | GGACTTCACCACGTACTGC |
| huTRAV26-1ext | GCAAACCTGCCTTGTAATC |
| huTRAV26-2ext | AGCCAAATTCAATGGAGAG |
| huTRAV27ext | TCAGTTTCTAAGCATCCAAGAG |
| huTRAV29ext | GCAAGTTAAGCAAAATTCACC |
| huTRAV30ext | CAACAACCAGTGCAGAGTC |
| huTRAV34ext | AGAACTGGAGCAGAGTCCTC |
| huTRAV35ext | GGTCAACAGCTGAATCAGAG |
| huTRAV36ext | GAAGACAAGGTGGTACAAAGC |
| huTRAV38ext | GCACATATGACACCAGTGAG |
| huTRAV39ext | CTGTTCCTGAGCATGCAG |
| huTRAV40ext | GCATCTGTGACTATGAACTGC |
| huTRAV41ext | AATGAAGTGGAGCAGAGTCC |
| huTRACext | GACCAGCTTGACATCACAG |
| huTRBV2ext | TCGATGATCAATTCTCAGTTG |
| huTRBV3ext | CAAAATACCTGGTCACACAG |
| huTRBV4ext | TCGCTTCTCACCTGAATG |
| huTRBV5-1_4ext | GATTCTCAGGKCKCCAGTTC |
| huTRBV5-5_8ext | GTACCAACAGGYCCTGGGT |
| huTRBV6-1_3,5_9ext | ACTCAGACCCCAAAATTCC |
| huTRBV6-4ext | ACTGGCAAAGGAGAAGTCC |
| huTRBV7-1_3ext | TRTGATCCAATTTCAGGTCA |
| huTRBV7-4_9ext | CGSWTCTYTGCAGARAGGC |
| huTRBV9ext | GATCACAGCAACTGGACAG |
| huTRBV10-1ext | CAGAGCCCAAGACACAAG |

| | |
|---|---|
| huTRBV10-2ext | ACCTTGATGTGTCACCAGAC |
| huTRBV11ext | CGATTTTCTGCAGAGACGC |
| huTRBV12ext | ARGTGACAGARATGGGACAA |
| huTRBV13ext | AGCGATAAAGGAAGCATCC |
| huTRBV14ext | CCAACAATCGATTCTTAGCTG |
| huTRBV15ext | AGTGACCCTGAGTTGTTCTC |
| huTRBV16ext | GTCTTTGATGAAACAGGTATGC |
| huTRBV17ext | CAGACCCCCAGACACAAG |
| huTRBV18ext | CATAGATGAGTCAGGAATGCC |
| huTRBV19ext | AGTTGTGAACAGAATTTGAACC |
| huTRBV20ext | AAGTTTCTCATCAACCATGC |
| huTRBV23ext | GCGATTCTCATCTCAATGC |
| huTRBV24ext | CCTACGGTTGATCTATTACTCC |
| huTRBV25ext | ACTACACCTCATCCACTATTCC |
| huTRBV27,28ext | TGGTATCGACAAGACCCAG |
| huTRBV29ext | TTCTGGTACCGTCAGCAAC |
| huTRBV30ext | TCCAGCTGCTCTTCTACTCC |
| huTRBCext | TAGAACTGGACTTGACAGCG |
| huTRAV1int | GCACCCACATTTCTKTCTTAC |
| huTRAV2int | CACTCTGTGTCCAATGCTTAC |
| huTRAV3int | ATGCACCTATTCAGTCTCTGG |
| huTRAV4int | ATTATATCACGTGGTACCAACAG |
| huTRAV5int | TACACAGACAGCTCCTCCAC |
| huTRAV6int | TGGTACCGACAAGATCCAG |
| huTRAV7int | TATGAGAAGCAGAAAGGAAGAC |
| huTRAV8-1int | GTCAACACCTTCAGCTTCTC |
| huTRAV8-3int | TTTGAGGCTGAATTTAAGAGG |
| huTRAV8-2,4int | AGAGTGAAACCTCCTTCCAC |
| huTRAV8-6int | AACCAAGGACTCCAGCTTC |
| huTRAV8-7int | ATCAGAGGTTTTGAGGCTG |
| huTRAV9-1_2int | GAAACCACTTCTTTCCACTTG |
| huTRAV10int | GAAAGAACTGCACTCTTCAATG |
| huTRAV12-1_3int | AAGATGGAAGGTTTACAGCAC |
| huTRAV13-1int | TCAGACAGTGCCTCAAACTAC |
| huTRAV13-2int | CAGTGAAACATCTCTCTCTGC |
| huTRAV14int | AGGCTGTGACTCTGGACTG |
| huTRAV16int | GTCCAGTACTCCAGACAACG |
| huTRAV17int | CCACCATGAACTGCAGTTAC |
| huTRAV18int | TGACAGTTCCTTCCACCTG |
| huTRAV19int | TGTGACCTTGGACTGTGTG |
| huTRAV20int | TCTGGTATAGGCAAGATCCTG |
| huTRAV21int | AACTTGGTTCTCAACTGCAG |
| huTRAV22int | CTGACTCTGTGAACAATTTGC |
| huTRAV23int | TGCATTATTGATAGCCATACG |
| huTRAV24int | TGCCTTACACTGGTACAGATG |
| huTRAV25int | TATAAGCAAAGGCCTGGTG |
| huTRAV26-1int | CGACAGATTCACTCCCAG |
| huTRAV26-2int | TTCACTTGCCTTGTAACCAC |
| huTRAV27int | CTCACTGTGTACTGCAACTCC |
| huTRAV29int | CTGCTGAAGGTCCTACATTC |
| huTRAV30int | AGAAGCATGGTGAAGCAC |
| huTRAV34int | ATCTCACCATAAACTGCACG |
| huTRAV35int | ACCTGGCTATGGTACAAGC |
| huTRAV36int | ATCTCTGGTTGTCCACGAG |
| huTRAV38int | CAGCAGGCAGATGATTCTC |

| | |
|---|---|
| huTRAV39int | TCAACCACTTCAGACAGACTG |
| huTRAV40int | GGAGGCGGAAATATTAAAGAC |
| huTRAV41int | TTGTTTATGCTGAGCTCAGG |
| huTRACint | TGTTGCTCTTGAAGTCCATAG |
| huTRBV2int | TTCACTCTGAAGATCCGGTC |
| huTRBV3int | AATCTTCACATCAATTCCCTG |
| huTRBV4int | CCTGCAGCCAGAAGACTC |
| huTRBV5-1_4int | CTTGGAGCTGGRSGACTC |
| huTRBV5-5_8int | TCTGAGCTGAATGTGAACG |
| huTRBV6-1_3,5_9int | GTGTRCCCAGGATATGAACC |
| huTRBV6-4int | TGGTTATAGTGTCTCCAGAGC |
| huTRBV7-1_3int | TCYACTCTGAMGWTCCAGCG |
| huTRBV7-4_9int | TGRMGATYCAGCGCACA |
| huTRBV9int | GTACCAACAGAGCCTGGAC |
| huTRBV10-1int | TGGTATCGACAAGACCTGG |
| huTRBV10-3int | GGAACACCAGTGACTCTGAG |
| huTRBV11int | GACTCCACTCTCAAGATCCA |
| huTRBV12int | CYACTCTGARGATCCAGCC |
| huTRBV13int | CATTCTGAACTGAACATGAGC |
| huTRBV14int | ATTCTACTCTGAAGGTGCAGC |
| huTRBV15int | ATAACTTCCAATCCAGGAGG |
| huTRBV16int1 | CTGTAGCCTTGAGATCCAGG |
| huTRBV17int | TGTTCACTGGTACCGACAG |
| huTRBV18int | CGATTTTCTGCTGAATTTCC |
| huTRBV19int | TTCCTCTCACTGTGACATCG |
| huTRBV20int | ACTCTGACAGTGACCAGTGC |
| huTRBV23int | GCAATCCTGTCCTCAGAAC |
| huTRBV24int | GATGGATACAGTGTCTCTCGA |
| huTRBV25int | CAGAGAAGGGAGATCTTTCC |
| huTRBV27,28int | TTCYCCCTGATYCTGGAGTC |
| huTRBV29int | TCTGACTGTGAGCAACATGAG |
| huTRBV30int | AGAATCTCTCAGCCTCCAGAC |
| huTRBCint | TTCTGATGGCTCAAACACAG |
