# Supplementary material for: Challenging immunodominance of influenza-specific CD8+ T cell responses restricted by the risk-associated HLA-A*68:01 allomorph
Source: Nat Commun. 2019 Dec 6;10:5579. doi: 10.1038/s41467-019-13346-4 (PMC6898063; doi:10.1038/s41467-019-13346-4)
Supplement: Supplementary file 2 — Reporting Summary [file 41467_2019_13346_MOESM2_ESM.pdf]

Editorial Note: Parts of this Peer Review File have been redacted as indicated to remove third-party material where no permission to publish could be obtained.

Reviewers' Comments:

Reviewer #1:
Remarks to the Author:
Following two papers that reported HLA A*6801 as a potential risk factor for severe influenza disease, the authors identified studied HLA A*6801 restricted influenza T-cell responses. The paper describes an in-depth characterization of a novel HLA A*6801 restricted influenza epitope that has several unique characteristics: (1) the bound peptide is 12 amino acids long, an extreme length for HLA class I epitopes; (2) Only 35% of the A*6801 positive donors studied produced an immunodominant response to this peptide; (3) The limited number of observed mutations in this epitops in natural influenza isolates which also exhibit temporal patterns of fixation do not contribute to T-cell escsape; (4) The antigen specific TCR repertoires of subjects exhibited relatively high diversity; and (5) differences in the T-cel phenotypes were observed between low and med/high responders.

Overall, this is very exciting paper, that studies a unique influenza specific T-cell response restricted by a risk associated allele, providing functional insights into the potential underlying mechanisms that may be associated with increased risk of severe disease in A*6801 individuals. The most important finding in this respect is simply the fact that the majority of A*6801 individuals fail to make a dominant robust response to this epitope, despite its very restricted ability to mutate, and the fact that the mutations that do appear do not contribute to T-cell escape. This suggests, that the majority of A*6801 positive individuals mount an overall weaker T-cell response to influenza, which could have important implications in pandemic settings, where antibodies cannot provide adequate protection.

However, several other findings in the paper, increase its importance and impact, and provide insights into the complex interactions between peptide-MHC and TCR. First, the fact that this peptide is 12 amino acids long. While there have been several previous reports of MHC bound peptides of this length, this is still a less explored and rare population of peptides. Second, given this unusual length, the finding that the antigen specific TCR repertoire is so diverse in most donors is also surprising. Finally, the T-cell phenotype analysis using the index-sorted cells provides insights into the functional differnces between the T-cells of the low vs. med/high responders, showing that a low response can be mediated by naive-like cells.

I think this is an important and ground breaking study. However, there are several additional analyses that I think may improve the paper. Furthermore, some of the figures require additional work as well.

Major comments:

1. Figure 3b and f - it seems that there is trending (p=0.058) association between the responses of the low vs. med/high responders, suggesting that perhaps the low responders to A*68 may actually be low responders overall. I would suggest plotting the correlation betwen the A*68 response and the overall magnitude of response to influenza when stimulating with a pool of epitopes. My guess is that there would be a significant correlation. This would be interesting, because its clear from the paper that there is great diversity in the response to this specific epitope, but the reason remain unclear. While the authors hypothesize that perhpas repeated boosting may be required to increase the responses in the low responders, another possible cause may be underlying genetic factors that may reduce their ability to mount an effective influenza (or more general) T-cell response. For example, if the other alleles these individuals carry do not bind dominant epitopes, the overall level of activation may be lower, leading to reduce and more naive-like responses. Since so much work has been done on characterizing the T-cell response of the subjects here, I would urge the authors to dig deeper into this data, going beyond the A*6801 specific response in order to explore other possible hypotheses leading to the majority of subjects generating low responses to this specific epitope.

2. Figure 7 and 8 require additional work and are not on par with the other figures in the paper. The facs plots are very hard to read, consider enlarging, or adding percentages for high vs. low in each plot. I would suggest drawing logo plots for the different clones based on sequence clustering. This can be done with many packages, and in particular with the TCRdist package used in the paper. Its not clear how the tables in the two figures differ one from another, they each have additional columns but also shared columns, so its unclear why the should be separated from one another? Perhaps instead one can use a single table with more columns, and replace the sequences with logo plots instead (Sequences can be added to supp).

3. Donor 7 is a very interesting outlier in several aspects, as seen in Figure 7. First the majority of his repertoire is based on two expanded TCRs. However, as can be seen in Figure 7, specific TCRs from the same Va, Vb and CDR3a/b appear as med/high and low responders. This suggests that even when an individual can mount a robust and memory like response to the A*68 epitope studied here, some of the clones that are closely related have varying degrees of response. Perhaps a more detailed comparison of some of these clones could shed light on the specific sequence motifs, or anchor positions that affect the reponse.

4. Donor 13 is also very interesting - From figure 3b we see that he only really responds to one of all of the p-MHC tetramers tested, but is the 2nd highest responder to the A*68 epitope. Interestingly, his other class I alleles are not ones which were tested as one of the other tetramers used in the study, as his alleles are not common alleles. He was also recruited in 2010, a year after the 2009 pandemic occured. It would be very interesting to dive deeper into his repertoire similarly to what was done in Figures 7/8 for the 3 other donors if that is possible. In particular, following the previous point, it would be interesting to test if he also has a diverse repertoire of clones from the same A-B pairing that exhibit a wide range of functional specificities - i.e. is this another case similar to donor 7, or is there something differnet happening here?

5. The paragraph in lines 305-313 is hard to follow and I could not make sense of what the argument was there? Please clarify.

6. The fact that the rare but fixted mutations in this epitope do not contribute to T-cell escape from this epitope are surprising. However, one may wonder whether these changes mediate esacpe from another, perhaps more potent epitope in this specific region. Pervious data from HIV has shown that epitopes tend to cluster in specific hostpots - which include several peptides that overlap one another. It would be great to use some off-the-shelf MHC binding predictor (e.g. IEDB) to identify all known epitopes that encompass this region and to use the same prediction method to test if these mutations provide escape from these other HLA restricted peptides. This may also help to identify additional epitopes in this region that may have not been tested using teteramers, which were selected for dominant epitopes identified in previous studies.

Minor comments:

1. Figure lengends overall can be improved. They are sometimes too short and do not fully explain the figure.

2. Figure 5b/c present data on 9 donors, but the text analyzes 8 of these.

3. Typo - line 289 to --> two

Reviewer #2:
Remarks to the Author:
In this manuscript, the authors performed a series of experiments to deeply investigate the features of human HLA-A*6801 specific T-cells. The flexible conformation of the peptide NP145 was revealed through the structure investigation. And this may be related with the diverse TCR repertoire in lower responder and medium/high responder. These newly investigated data are crucial for the understanding of the HLA-A*6801 specific T-cells and its susceptibility to influenza.

1. Title: the word "potential" may be not suitable. Title should describe the conclusion based on the facts that occurred.
2. The 35% in the conclusion may be not suitable considering the low sampling scale covering only 17 donors.
3. Page 6, Line 174, when you determine the cross-reactivity of the human T cells to the peptides and its different substitutes, the background of the donors should be investigated. Was the donor infected by both the 146A and 146T viruses? This is very important to explain the cross-reactivity.
4. Page 6, Line177, the description about "escape" may be not the right word herein. The mutation may be due to the fitness or the re-assortment of the virus.
5. Page 6 Line 194, the cutoff for the low responders and the medium responders should have the same cutoff (maybe 20), not the current different cutoffs, such as <12 for low responder and >20 for medium.
6. Page 7 Line 221, based on the figures, "3 out of 4" should be 5 out of 6. Line 224, Donor number 6 should be 7. Line 225, 6 donors and Line 226, 4 donors should be both 7 donors.
7. Is there any bias if the TCR repertoire was amplified in vitro before testing. And also for the memory phenotype?
8. Is it trustable for the results of the HLA typing? It is very important to confirm the HLA typing of the donors through DNA sequencing.
9. Figure 2b, were all the cells in this figure cultured 10 days before test? What is the stimulus if the cells were cultured. For the " A68/NP 145-PE",is it the tetramer for the test of the results? Why were there so many positive cells for DMSO control when staining the cells with tetramer?
10. Figure 2c, what is the result if the cells were cultured with 146V peptide before testing.

Reviewer #3:
Remarks to the Author:
This well written and executed study by van de Sandt and colleagues assesses the CD8 T cell response to a non classical conserved influenza peptide presented by HLA A68:01. The authors demonstrate the structure of the peptide in MHC to high resolution revealing flexibility in the peptide where TCR contacts are likely to occur. They demonstrate heterogeneity in responses across donors and find that memory phenotype correlates with the level of cells in the blood. Finally by single cell TCR sequencing they find expanded clones in the med/high responders which potentially suggest that it requires multiple exposures to expand this population of Ag-specific T cells.

One concern with this study is the low number of total donors. While it would not be necessary to repeat all of the analyses it would greatly strengthen the conclusions to increase the number of patients sampled for responder (high med low) and memory phenotype.

It would also be interesting to include any recent vaccination history information. While it is difficult to determine all previous exposures, it may be possible to correlate recent vaccine with the level of response/memory to this epitope.

Finally given the breadth of conserved antigens CD8 T cells respond to during influenza infection it is unclear how the dynamics of this one epitope will impact protection/reduction from disease severity.

RESPONSES TO REVIEWERS' COMMENTS

We thank the Reviewers for their constructive comments and appreciation for the importance of our study: "I think this is an important and ground breaking study." (Reviewer 1); "These newly investigated data are crucial for the understanding of the HLA-A*6801 specific T-cells and its susceptibility to influenza." (Reviewer 2); "This well written and executed study by van de Sandt and colleagues assesses the CD8 T cell response to a non classical conserved influenza peptide presented by HLA A68:01." (Reviewer 3).

We responded to Reviewer's questions and comments in a point-by-point form.

We appreciate the opportunity to re-submit our study to *Nature Communications* and are happy to make any additional modifications suggested by the Editors and Reviewers.

Reviewer #1 (Remarks to the Author):

Major comments:

*1. Figure 3b and f - it seems that there is trending (p=0.058) association between the responses of the low vs. med/high responders, suggesting that perhaps the low responders to A*68 may actually be low responders overall. I would suggest plotting the correlation between the A*68 response and the overall magnitude of response to influenza when stimulating with a pool of epitopes. My guess is that there would be a significant correlation. This would be interesting, because its clear from the paper that there is great diversity in the response to this specific epitope, but the reason remain unclear. While the authors hypothesize that perhaps repeated boosting may be required to increase the responses in the low responders, another possible cause may be underlying genetic factors that may reduce their ability to mount an effective influenza (or more general) T-cell response. For example, if the other alleles these individuals carry do not bind dominant epitopes, the overall level of activation may be lower, leading to reduce and more naive-like responses. Since so much work has been done on characterizing the T-cell response of the subjects here, I would urge the authors to dig deeper into this data, going beyond the A*6801 specific response in order to explore other possible hypotheses leading to the majority of subjects generating low responses to this specific epitope.*

We thank the Reviewer for this this sharp observation. We have made the same observation when we initially analyzed the data presented in Figure 3b, where we noticed that the overall frequency of universal epitope-specific $CD8^+$ T cells in the A68/NP low responders might be lower as compared to the medium and high responders. We therefore analysed our data in several ways to determine whether this trend was indeed significant, as observed for the $A68/NP_{145}$-specific $CD8^+$ T cells:

1) The overall frequencies of universal epitope-specific $CD8^+$ T cells in our low responders was significantly higher when compared to the $A68/NP_{145}$ frequency (Figure 3c).

2) The overall frequencies of universal epitope-specific $CD8^+$ T cells in our medium/high responders was similar to the $A68/NP_{145}{}^+CD8^+$ T cells frequency in our medium/high responders (Figure 3d).

3) The overall $A68/NP_{145}{}^+CD8^+$ T cell response in our low responders was significantly lower when compared to our medium/high responders (Figure 3e).

4) Although there is a trend that the overall universal $CD8^+$ T cell response in our low responders was lower when compared to the medium/high responders, this was not significant (Figure 3f).

5) Following the Reviewer's suggestion, we also correlated the $A68/NP_{145}{}^+CD8^+$ T cell frequency against the universal epitope-specific $CD8^+$ T cell frequencies. Although we observed a trend, no significant correlation was found (please see the figure below). Based on the Reviewer's suggestion, we have now added this figure as a supplemental figure to the manuscript (Supplemental Fig. 3)

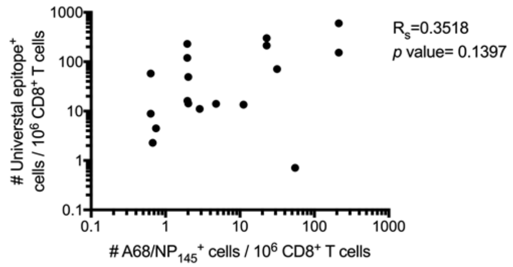

6) We did not observe a more naïve-like phenotype in the universal epitope-specific $CD8^+$ T cell response for our low responding donors (Figure 4d).

So, although there was a trend, we could not find any statistical evidence that the overall universal epitope-specific $CD8^+$ T cell responses were lower in low responders versus the medium/high responders, even when the data were analysed in several ways.

Following the Reviewer's comment, we have included the following sentence in the manuscript (Line 211-216):

"*Although there was a trend for a lower overall $CD8^+$ T cell frequency directed at the universal influenza epitopes in the low responders, as compared to the medium and high responders, this was not significant (p=0.058, Fig. 3f). Additionally, no correlation was found between the frequency of $A68/NP145^+CD8^+$ T cells and the frequency of $CD8^+$ T cells directed against the universal epitopes (Supplemental Fig. 3).*"

Supplemental Figure 3 legend:

"***Supplemental Figure 3. $A68/NP_{145}{}^+CD8^+$ T cell frequencies do not correlate with universal epitope-specific $CD8^+$ T cell frequencies.***

A correlation analysis between the frequency of A68/NP_{145}-specific $CD8^+$ T cells and universal epitope-specific $CD8^+$ T cells (n=13) was performed using Spearman's correlation coefficient (r_S)."

2. Figure 7 and 8 require additional work and are not on par with the other figures in the paper. The facs plots are very hard to read, consider enlarging, or adding percentages for high vs. low in each plot. I would suggest drawing logo plots for the different clones based on sequence clustering. This can be done with many packages, and in particular with the TCRdist package used in the paper. Its not clear how the tables in the two figures differ one from another, they each have additional columns but also shared columns, so its unclear why the should be separated from one another? Perhaps instead one can use a single table with more columns, and replace the sequences with logo plots instead (Sequences can be added to supp).

We thank the Reviewer for this constructive comment.

We have now increased the size, dots and/or lines of the FACS plots in Figure 2b, 3a, 4b, 7 and Sup Fig 1.

We have again analysed the data with TCRdist package, both total TCR repertoire, the expanded repertoire and TCR repertoire based on the phenotype of index sorted cells. Unfortunately, neither of these TCRdist analysis created logo plots or CDR3 motifs. This is most likely due to the high variety of both expanded and non-expanded TCRs within and between donors.

We acknowledge the Reviewer's suggestion to replace these tables with logo plots. However, our aim for representing the data in Figures 7 and 8 was to link MFIs of particular TCRαβ clonotypes (reflecting tetramer binding affinity) or phenotypes to TCRαβ signatures respectively. This was feasible as we performed single-cell index sorting and to the best of our knowledge, such data have not been published to date.

Our main findings related to these data are:

- Expanded clonotypes observed in medium and high responding donors but not low responding donors (Table 2 and Figure 6)
- Clonotype distribution based on tetramer affinity (Figure 7 (including table + MFI))
- Expanded clonotypes being of a memory phenotype (Figure 8 (including tables))

Furthermore, a single TCR table including all the TCR data can be found in Supplementary Table 3.

We would also like to highlight that these tables also contain complementary information.

Table 2 is a shorter version of supplementary table 3, containing the expanded clonotypes.

Figure 7: Each table contains index-sorted clonotypes of a single donor. The clonotypes are ordered based on the tetramer MFI from their respective cell (high to low)

Figure 8: These tables only contain the directly *ex vivo* index-sorted donors, each table contains clonotypes of cells that belong to a different phenotype e.g. Tcm, Teff, Temra, Tnaive, Tscm.

*3. Donor 7 is a very interesting outlier in several aspects, as seen in Figure 7. First the majority of his repertoire is based on two expanded TCRs. However, as can be seen in Figure 7, specific TCRs from the same Va, Vb and CDR3a/b appear as med/high and low responders. This suggests that even when an individual can mount a robust and memory like response to the A*68 epitope studied here, some of the clones that are closely related have varying degrees of response. Perhaps a more detailed comparison of some of these clones could shed light on the specific sequence motifs, or anchor positions that affect the response.*

We thank the Reviewer for this suggestion.

We agree with the Reviewer that Donor 7 is very interesting. Indeed, the majority of the TCRαβ repertoire comprises of one expanded TCRαβ clonotype TRBV-CASSSPSGVYNEQ and TRAV-CLVGDLINSGGYNKLIF, and a number of smaller clonotypes. The dominant clonotype can be found across higher and lower MFIs. This indicates that the MFI of tetramer binding might not only be affected by TCRαβ segments but also most probably by TCR levels, TCR dynamics, TCR spatial arrangements and other intrinsic factors.

We have included the above observation in the manuscript (line 309-315):

"*Interestingly, in donor 7 the majority of the TCRαβ repertoire comprises of one expanded TCRαβ clonotype TRBV6-6-CASSSPSGVYNEQ and TRAV4-CLVGDLINSGGYNKLIF, and a number of smaller clonotypes (Fig.7). The dominant clonotype was found across higher and lower MFIs. This indicates that MFI of tetramer binding might be not only affected by TCRαβ chains but also most probably TCR levels, TCR dynamics, TCR spatial arrangements and/or other intrinsic factors.*"

We have also enlarged the individual dots in the FACS plots of Figure 7.

Furthermore, we plan to perform a more detailed comparison of some of these clones by structural analyses.

*4. Donor 13 is also very interesting - From figure 3b we see that he only really responds to one of all of the p-MHC tetramers tested, but is the 2nd highest responder to the A*68 epitope. Interestingly, his other class I alleles are not ones which were tested as one of the other tetramers used in the study, as his alleles are not common alleles. He was also recruited in 2010, a year after the 2009 pandemic occured. It would be very interesting to dive deeper into his repertoire similarly to what was done in Figures 7/8 for the 3 other donors if that is possible. In particular, following the previous point, it would be interesting to test if he also has a diverse repertoire of clones from the same A-B pairing that exhibit a wide range of functional specificities - i.e. is this another case similar to donor 7, or is there something differnet happening here?*

We agree with the Reviewer that the response observed in donor 13 was also interesting. This was one of the initial donors recruited for our study. Figure 3b shows that this donor is indeed one of the highest responders, second highest frequency of A68/NP_{145} observed. Unfortunately, we were unable to compare the A68/$NP_{145}$$^{+}$$CD8^{+}$ T cells to $CD8^{+}$ T cell responses directed against other epitopes as the influenza epitopes associated with other HLAs in this donor are currently unknown.

Since Donor 13 was a patient hospitalized with influenza disease, we had only two PBMC vials of this donor available, and no opportunity for follow up due to the nature of our ethics. We used one vial to establish the frequency and phenotype (Fig 3 and Fig 4), and the second vial for TCR analysis (Fig 5). To obtain a sufficient number of A68/NP_{145}-specific $CD8^{+}$ cells from this donor for single cell TCR analysis, we needed to expand the A68/NP_{145}-specific $CD8^{+}$ cells *in vitro*

As the Reviewer correctly observed, this donor was recruited in 2010. Although the influenza type was not specified, the donor was most likely infected with the H1N1pdm09 strain or the H3N2 strain (two influenza A strains circulating after 2009). Both A/H3N2 and A/H1N1pdm09 contain the D**A**TYQRTRALVR variant of the epitope, which was also used for our tetramer analysis, which confirms that the donor responded to this epitope during influenza virus infection.

5. The paragraph in lines 305-313 is hard to follow and I could not make sense of what the argument was there? Please clarify.

We thank the Reviewer for this comment.

We have now rephrased this paragraph (line 316-325), so it reads:

*"Subsequently, we dissected the A68/NP145^{+}$CD8^{+}$ TCR$\alpha\beta$ repertoires of all index-sorted donors (1b, 2b, 3b, 6 and 7) according to their matched phenotypes (Fig. 8). Here, we focused on the commonly observed clonotypes TRBV20-1 and TRAV4 (shared between donors 1b, 2b, 3b, 5, 6, 7 and 16) and the donor-specific clonotypes TRBV30 (donor 6) and TRBV6-6 (donor 7), which were detected at relatively high frequencies in those donors. We found that the expanded TCR$\alpha\beta$s within common gene segments (TRBV20-1, TRAV4) and the high frequency individual clonotypes (TRBV30 and TRBV6-6) were highly prevalent in the memory $CD8^{+}$ T cell populations (Fig. 8). These results confirm that the large TCR$\alpha\beta$ clonal expansions observed within the medium and high HLA-A*68:01-responding donors were predominantly of the memory phenotype (Fig. 6, Fig. 8)."*

6. The fact that the rare but fixted mutations in this epitope do not contribute to T-cell escape from this epitope are surprising. However, one may wonder whether these changes mediate escape from another, perhaps more potent epitope in this specific region. Pervious data from HIV has shown that epitopes tend to cluster in specific hostpots – which include several peptides that overlap one another. It would be great to use some off-the-shelf MHC binding predictor (e.g. IEDB) to identify all known epitopes that encompass this region and to use the same prediction method to test if these mutations provide escape from these other HLA restricted peptides. This may also help to identify additional epitopes in this region that may have not been tested

using tetramers, which were selected for dominant epitopes identified in previous studies.

We agree with the Reviewer that this mutation may provide escape for another influenza A peptide binding a different HLA molecule. Based on the Reviewer's suggestion, we have searched the IEDB database for other NP-peptides and aligned those with the NP protein. This resulted in 4 overlapping peptides, namely $NP_{146-154}$ (HLA-A*02:03, 68:02, HLA-B*14:02, 02:02 and HLA-C*06:02), $NP_{140-148}$ (HLA-A*01:01,26:01, 30:02, 80:01, HLA-B*15:01, 15:17, 35:01, 57:01, 58:01) $NP_{140-150}$ (HLA-B*15:01) and $NP_{139-156}$ (HLA-B*15:01). The overlapping peptides in the IEDB all have an Ala at position 146, except for $NP_{146-154}$ which has a Thr at position 146. It is possible that the Thr or Val variants at position 146 result in T cell escape for some, if not all, of these other peptides-specific $CD8^+$ T cell responses. We have further clarified this in the Discussion:

Line 334-337 now reads:

"*We found that the NP_{145} viral peptide was highly conserved across influenza strains, except for position 146 (P2 anchor residue position of the peptide), although this did not result in viral escape from the A68/NP_{145}-specific $CD8^+$ T cell response*."

We have also added the following sentence in the Discussion (line 369-375):

"*However, according to the IEDB database (www.iedb.org), the $NP_{145-156}$ peptide overlaps with at least 4 other peptides, namely* $NP_{146-154}$ (HLA-A*02:03, 68:02, HLA-B*14:02, 02:02 and HLA-C*06:02), $NP_{140-148}$ (HLA-A*01:01,26:01, 30:02, 80:01, HLA-B*15:01, 15:17, 35:01, 57:01, 58:01) $NP_{140-150}$ (HLA-B*15:01) and $NP_{139-156}$ (HLA-B*15:01)*. It is thus possible that the variation observed at position NP_{146} is driven by the virus' ability to escape from $CD8^+$ T cells responses directed against one or more of these overlapping peptides instead*."

Minor comments:

1. Figure legends overall can be improved. They are sometimes too short and do not fully explain the figure.

We thank the Reviewer for this suggestion. We have modified figure legends by providing more details.

2. Figure 5b/c present data on 9 donors, but the text analyses 8 of these.

We acknowledge the Reviewer's comment that this figure may lead to confusion for the reader. We had depicted the 2015 and 2018 TCR repertoire of donor 3 in two separate pie charts in Figure 5b. To avoid any confusion on the number of donors in this figure, we have now combined the two pie charts for this donor in one pie chart, similar as to what we had already done for the circus plot (Figure 5c) and the CDR3 lengths (Figure 5d).

3. Typo - line 289 to --> two

We have corrected the typo.

Reviewer #2 (Remarks to the Author):

1. Title: the word "potential" may be not suitable. Title should describe the conclusion based on the facts that occurred.

We thank the Reviewer for this suggestion. We have now changed the title according to the Reviewer's suggestion, which now reads:

"*Challenging immunodominance of influenza-specific CD8$^+$ T cell responses directed against the risk-associated HLA*68:01 allomorph*"

2. The 35% in the conclusion may be not suitable considering the low sampling scale covering only 17 donors.

Our study tested influenza-specific CD8$^+$ T cell responses in 18 HLA-A*68:01-expressing donors. Our 18 donors assessed in our study represent all the HLA-A*68:01 individuals recruited and HLA typed across 6 different cohorts, consisting of ~500 donors, which took over a decade to establish. Screening donors for the HLA-A*68:01 allele requires full HLA typing by the Australian Red Cross Blood Service (ARCBS) of all potential donors in the cohort, which is a time-consuming and costly ($AUD 250 per donor; total cost for these 18 donors out of 500 individuals was $AUD 125,000). Thus, we hope the Reviewer appreciates the uniqueness of our study dissecting influenza-specific CD8$^+$ T cell responses in these 18 HLA-A*68:01 donors.

Unfortunately, HLA-A*68:01 is a rare HLA allomorph, with the highest frequencies in populations living in South America. The percentage of HLA-A*68:01 allele in the Australian population is 4.1%, which is based on our own screenings of 6 Australian cohorts and information from the HLA database (http://www.allelefrequencies.net/). This means that to recruit 1 new HLA-A*68:01-expressing donor for our study, we would have to recruit and HLA type additional 25 healthy donors, which as mentioned above, would be both costly and time-consuming.

Following the Reviewer's comment, we added a sentence in Methods to explain the number of HLA-A*68:01-expressing donors and our cohorts (line 435-437):

"*Our study assessed influenza-specific CD8$^+$ T cell responses in 18 HLA-A*68:01-expressing donors. These 18 donors represent all of our HLA-A*68:01 individuals recruited and HLA typed across 6 different HLA-typed cohorts, consisting of a total of ~500 donors.*"

3. Page 6, Line 174, when you determine the cross-reactivity of the human T cells to the peptides and its different substitutes, the background of the donors should be investigated. Was the donor infected by both the 146A and 146T viruses? This is very important to explain the cross-reactivity.

We thank the Reviewer for this constructive comment. We have a great expertise studying $CD8^+$ T cell cross-reactivity in human donors and therefore agree with the importance of exposure history for cross-reactivity between different peptide variants (e.g. *van de Sandt 2014 J Virol, Quinones-Parra 2014 PNAS, van de Sandt 2015 J Gen Virol)*. Influenza exposure history could, to a certain extent, be examined by extensive serological analysis, which aims to link antibody titres to historic influenza virus strains within these donors, as previously described in *van de Sandt 2015 JID*. Unfortunately, we do not have access to serum samples for these donors prohibiting analysing these history antibody responses.

However, based on the age of the donors we have a reason to believe all donors were exposed to viruses expressing both the 146A and 146T variant of the peptide. Donor 6 was born in 1987, donor 18 was born in 1991. Although we have no exact day of birth for donor 15, we know the donor was recruited in 2017 and would have been 18 years or older at time of recruitment, this means that the donor was born before 1999. Two independent studies have demonstrated that by the age of 3 years 80% of the children would have experienced at least one influenza A virus infection, increasing to 100% by the age of 7 (*Bodewes 2011 Clin Vac Imm* and *Sauerbrei 2014 Euro Surv).* By this logic, we can assume that all three donors would have been infected with an influenza A virus expressing the 146T variant of the peptide, since A/H3N2 and A/H1N1 strains circulating prior to 2001 expressed the 146T variant (line 158-167). Even though influenza virus infection are less frequently observed in adults as in children, adults still encounter 2 influenza virus infections per decade (*Kucharski 2015 Plos Biol)*. It is therefore reasonable to assume that all 3 donors would have had at least one additional influenza virus infections after 2001 with either the A/H3N2 virus strain and/or the A/H1N1pdm09 strain both expressing the 146A variant of the peptide (line 161-167). The chance that these donors would have encountered a natural virus infection, which contained the 146V variant of the peptide is highly unlikely as this variant was only observed in 7 out of the 24408 human influenza A virus isolates recorded between 1918 and 2018.

To study cross-reactivity, we initially stimulated the PBMCs of those 3 donors with either the 146A or 146T variant of the peptide to expand the 146A or 146T-specific $CD8^+$ T cells, respectively (Fig. 2c middle and right panel). We have subsequently tested the cross-reactivity by re-stimulating with the 146A expanded cells with the 146T and 146V variant of the peptide and the 146T expanded cells with the 146A and 146V variant of the peptide, which in both cases resulted in a strong IFNy response similar to the response observed when cells were re-stimulated with the same peptide used for primary stimulation (fig 2b and c).

Following the Reviewer's comment, we added the following sentence to Discussion (Line 345-360):

"*Donors used to study the cross-reactivity of the A68/NP_{145}-specific $CD8^+$ T cell response were likely to have been exposed to viruses expressing both the 146A and 146T variant of the NP_{145} peptide. Donor 6 was born in 1987, donor 18 was born in 1991. Although the exact date of birth for donor 15 is unknown, the donor was recruited in 2017 and would have been 18 years or older at time of recruitment, hence born before 1999. It was demonstrated that by the age of 3, 80% of the children would have experienced at least one influenza A virus infection, increasing to 100% by the age of 7 (Bodewes 2011 Clin Vac Imm and Sauerbrei 2014 Euro*

Surv). Thus, all three donors would have been infected with an influenza A virus expressing the 146T variant of the peptide, which was expressed in A/H3N2 and A/H1N1 strains circulating prior to 2001. Even though influenza virus infection is less frequently observed in adults than in children, adults still encounter two influenza virus infections per decade (Kucharski 2015 Plos Biol). It is therefore reasonable to assume that all 3 donors would have had at least one additional influenza virus infections after 2001 with either the A/H3N2 virus strain and/or the A/H1N1pdm09 strain, both expressing the 146A variant of the peptide. The chance that these donors would have encountered the 146V variant of the peptide via natural infection is highly unlikely, as this variant was only observed in 7 out of the 24408 human influenza A virus isolates recorded between 1918 and 2018."

4. Page 6, Line177, the description about "escape" may be not the right word herein. The mutation may be due to the fitness or the re-assortment of the virus.

Following the Reviewer's comment, we have rephrased this sentence (line 178-182), so it now reads:

"*The high level of cross-reactivity between the 146A, 146T and 146V peptide variants suggests that fixation of these mutations at position 146 did not result in viral escape from pre-existing A68/$NP_{145}^{+}CD8^{+}$ T cell responses and would therefore not be a determining factor in HLA-A*68:01-associated morbidity when a new variant is introduced*."

5. Page 6 Line 194, the cutoff for the low responders and the medium responders should have the same cutoff (maybe 20), not the current different cutoffs, such as <12 for low responder and >20 for medium.

We thank the Reviewer for this suggestion. We have now changed the cut off to <12 for low responders and >12 for medium responders. More specifically we changed, line 202, Figure 3b and figure legend to Figure 3 and 4.

6. Page 7 Line 221, based on the figures, "3 out of 4" should be 5 out of 6. Line 224, Donor number 6 should be 7. Line 225, 6 donors and Line 226, 4 donors should be both 7 donors.

We acknowledge that this section was confusing. We would like to point out that we measured two time points for 3 out of the 4 low responding donors, namely donor 1, donor 2 and donor 3 (samples collected in 2015 (a, open circle) and samples collected in 2018 (b, closed circle)). Even though we had already indicated this in Table 1 and in the legend of Fig 2c by using the open and closed symbols, we have now made an extra note in the accompanying figure legend, which now reads:

"*We included two measurements for donor 1, 2 and 3: open symbols were used for samples collected in 2015, closed symbols for samples collected in 2018 (see also table 1).*"

We have included a total of 6 medium/high responders, donor 6 was measured in two independent experiments and was therefore included as two separate symbols. Average of the two measurements was used for statistics. We realize that this may

have caused some confusion and have therefore decided to only show the average of the two measurements, thus have changed Figure 3c, accordingly.

7. Is there any bias if the TCR repertoire was amplified in vitro before testing. And also for the memory phenotype?

We agree that *in vitro* expansion leads to different memory phenotypes, hence we have not added Donor 5, 16 and 13 to our phenotypic index-sorted analyses (Fig.8).

Furthermore, analysis of frequencies and phenotypes established in Figures 3 and 4, respectively were established directly *ex vivo*, without prior expansion of the A68/NP_{145}-specific $CD8^+$ T cells.

However, A68/NP_{145}-specific $CD8^+$ T cells of donor 5, 16 and 13 had to be expanded to obtain enough cells for single-cell sorting to establish the TCR repertoire*. In vitro* expanded donors were indicated by an asterisk in Figure 5 and 6 respectively.

Regarding TCRs, as shown by our previous study in CMV (Nguyen 2014 J Immunol) directly comparing TCR repertoires *ex vivo* and *in vitro* expanded, there is ~70% overlap between the clonotypes found in both repertoires (please see the table and fig below, bar graph). As we have sequenced only <35 clonotypes from single-cell sorted tetramer$^+$$CD8^+$ T cells, and TCR repertoire is a function of a sequencing depth, it is likely that sequencing more clonotypes would increase the overlap of TCRαβ clonotypes above ~70%.

Furthermore, analyses of TRAV and TRBV gene segments after ex vivo and *in vitro*-expansions shows comparable usage of predominant TRAV and TRBV genes (please see table below and the bar graph+pie charts)

[redacted table and figure from Nguyen 2014 J Immunol]

8. Is it trustable for the results of the HLA typing? It is very important to confirm the HLA typing of the donors through DNA sequencing.

We agree with the Reviewer and therefore are happy to confirm that all of our donors were fully 4-digit HLA typed via molecular genotyping performed by the Australian Red Cross Blood Service (ARCBS), accredited to perform HLA typing for bone marrow and organ transplantations in Australia.

We have now added the following sentence to the manuscript (line 444-445):

"*HLA class I and class II molecular genotyping was performed from genomic DNA by the ARCBS.*"

9. Figure 2b, were all the cells in this figure cultured 10 days before test? What is the stimulus if the cells were cultured. For the "A68/NP 145-PE",is it the tetramer for the test of the results? Why were there so many positive cells for DMSO control when staining the cells with tetramer?

Figure 2b shows representative FACS plots of a sample stimulated with the 146A peptide at day 0 and re-stimulated on day 10 with the stimulations mentioned on top of the FACSplots (PMA/Iono, DMSO, Pool, 146A, 146T or 146V, respectively).

We have included tetramer staining in day 10 staining panel to confirm that the A68/NP_{145}-specific $CD8^+$ T cells had expanded after initial stimulation using the peptide pool, or single 146A and 146T peptides at day 0.

We have clarified this in the legend to Figure 2, which now reads:

"*Representative FACS panels of A68/NP_{145}-specific $CD8^+$ T cells expanded by 146A peptide stimulation on day 0, followed by re-stimulation at day 10 with cognate or variant NP145 peptides. Day 10 re-stimulation with PMA/Ionomycin was included as a positive control and DMSO as a negative control.*"

The FACS plots are representatives of 146 peptide-stimulated and expanded A68/NP_{145}-specific $CD8^+$ T cells at day 10, hence the DMSO control was used for second re-stimulation (6 hrs) of these expanded cells. The large population of tetramer-positive cells was the result of the initial expansion after the first stimulation.

The second 6-hr stimulation was to induce a cytokine response, which occurred for the respective peptide stimulations and the positive control (PMA/Iono) but not for the DMSO negative control. The reason why we observed a higher percentage of tetramer positive cells in the DMSO group is probably because $CD8^+$ T cells tend to down regulate their TCR receptors after stimulation, hence the overall MFI and the frequency of the tetramer positive cells for the secondary peptide stimulated groups is lower as compared to the DMSO control.

We have included the following sentence in Results (line 171-176):

"*The FACS plots are representatives of 146A peptide-stimulated and expanded A68/NP_{145}-specific $CD8^+$ T cells at day 10. DMSO was used as a negative control for the second 6-hr re-stimulation in an IFN-γ ICS assay of these expanded cells, thus the large population of tetramer-positive cells resulted from the initial expansion after the first stimulation (Fig. 2b). However, negligible IFN-γ production was detected in the DMSO control (Fig. 2b).*"

10. Figure 2c, what is the result if the cells were cultured with 146V peptide before testing.

We agree with the Reviewer that in order to complete the picture on cross-reactivity between different peptide variants, we would ideally also have included a condition where stimulated PBMCs with the 146V variant on day 0.

Unfortunately, we were limited by the number of cells from HLA-A*68:01 donors available for this assay, which allowed us to only test 3 conditions. As explained in question 3, it is likely that all of our 3 donors have encountered the 146A and/or 146T variants of this peptide and highly unlikely that they have encountered the 146V variant. We therefore decided to specifically expand using the 146A and 146T-specific $CD8^+$ T cells to confirm their cross-reactivity and use the peptide pool (which included the 146V variant) as a positive control for the expansion of all A68/NP_{145}-specific $CD8^+$ T cells in these donors. Such comparable high response observed for the 146A and 146T expanded $CD8^+$ T cells versus the pool of expanded $CD8^+$ T cells indicates that high cross-reactivity of the A68/NP_{145}-specific $CD8^+$ T cells between the different variants of the peptide. So even though the donors would probably not have encountered a 146V variant via the natural influenza virus infection, it is very likely that an initial 146V peptide stimulation on day 0 would also expand the A68/NP_{146}-specific $CD8^+$ T cells displaying high cross-reactivity upon re-stimulation with the 146A and 146T variants of the peptide at day 10, as we observed for the 146A- and 146T-expanded $CD8^+$ T cells.

Reviewer #3 (Remarks to the Author):

1) One concern with this study is the low number of total donors. While it would not be necessary to repeat all of the analyses it would greatly strengthen the conclusions to increase the number of patients sampled for responder (high med low) and memory phenotype.

As we have responded to Reviewer 2 (question 2):

Our study tested influenza-specific $CD8^+$ T cell responses in 18 HLA-A*68:01-expressing donors. Our 18 donors assessed in our study represent all the HLA-A*68:01 individuals recruited and HLA typed across 6 different cohorts, consisting of ~500 donors, which took over a decade to establish. Screening donors for the HLA-A*68:01 allele requires full HLA typing by the Australian Red Cross Blood Service (ARCBS) of all potential donors in the cohort, which is a time-consuming and costly exercise ($AUD 250 per donor; total cost for these 18 donors out of 500 individuals was $AUD 125,000). Thus, we hope the Reviewer appreciates the uniqueness of our study comprising of those 18 HLA-A*68:01 donors.

Unfortunately, HLA-A*68:01 is a rare HLA allomorph, with the highest frequencies in populations living in South America. The percentage of HLA-A*68:01 allele in the Australian population is 4.1%, which is based on our own screenings of 6 Australian cohorts and information from the HLA database (http://www.allelefrequencies.net/). This means that to recruit 1 new HLA-A*68:01-expressing donor for our study, we would have to recruit and HLA type additional 25 healthy donors, which would be both costly and time-consuming.

Following the Reviewer's comment, we added a sentence in Methods to explain the number of HLA-A*68:01-expressing donors and our cohorts (line 435-437):

"*Our study assessed influenza-specific $CD8^+$ T cell responses in 18 HLA-A*68:01-expressing donors. These 18 donors represent all of our HLA-A*68:01 individuals recruited and HLA typed across 6 different HLA-typed cohorts, consisting of a total of ~500 donors.*"

2) It would also be interesting to include any recent vaccination history information. While it is difficult to determine all previous exposures, it may be possible to correlate recent vaccine with the level of response/memory to this epitope.

We agree with the Reviewer that both previous influenza virus exposure and vaccine history would be an interesting feature to include in our study.

Unfortunately, we were limited by the information provided by the donors during the consent. Unfortunately, the majority of our cohorts did not collect background information on infection history, with the exception for the cohort that recruited donor 11, 12 and 13 (see Table 1, specifications column). These donors were recruited upon admission to the hospital with a respiratory infection. Only donor 13 was infected with an influenza A virus at time of admission (Table 1), which could explain the high frequency of $A68/NP_{145}^+$ $CD8^+$ T cells observed in this donor.

The elderly cohort from which donor 7 was recruited, was the only cohort that had records of the influenza vaccination status. We can therefore confirm that donor 7 was vaccinated in the year prior to recruitment.

The remaining donors are healthy adults recruited in Australia. The Australian policy on influenza vaccination is on a voluntary basis, without special recommendations for healthy adults. Unfortunately, cohorts did not keep records on influenza vaccine status of those donors.

Furthermore, currently used seasonal influenza vaccines are predominantly inactivated vaccine preparations that aim at the induction of strain-specific antibodies but fail to boost the influenza virus-specific $CD8^+$ T cell repertoire (Koutsakos M, STM 2018, *Cox 2004 Scan J Immunol, Hoft 2011 JID, He 2006 J Virol, Basha 2011 Hum Immunol*). A potential influenza vaccination history (eg donor 7) would therefore not have resulted in an increased $A68/NP_{145}{}^+CD8^+$ T cell frequency, if anything inactivated influenza vaccines may hamper the development of a robust influenza virus-specific $CD8^+$ T cell response which would otherwise be boosted by natural infections (*Bodewes 2009 Lancet Inf Dis*).

Following the Reviewer's comment, we have added the following sentence to the modified version of the manuscript (Methods, page 452-455):

"*The vaccination and infection history of the donors were predominantly unknown. However, it is important to note that the current inactivated influenza vaccine does not induce influenza-specific $CD8^+$ T cells responses (Koutsakos M, Sci Transl Med, 2018), thus recent influenza immunisation would not affect $CD8^+$ T cell responses tested in this study."*

3) Finally given the breadth of conserved antigens CD8 T cells respond to during influenza infection it is unclear how the dynamics of this one epitope will impact protection/reduction from disease severity.

We agree with the Reviewer that a potential low $CD8^+$ T cell frequency against this one epitope may not greatly affect the disease severity in individuals who also express HLA allomorphs capable to present universal influenza epitopes and therefore mount a robust influenza $CD8^+$ T cell response against these epitopes. However, as the frequency of the HLA-A*68:01 allomorph is especially high among the Indigenous populations (http://www.allelefrequencies.net, Clemens ICB 2016), especially those living in Southern America and Australia, these Indigenous populations often lack HLA allomorphs that present universal influenza epitopes and thus might lack influenza virus-specific $CD8^+$ T cell responses towards other HLAs. The fact that the $A68/NP_{145}$-specific $CD8^+$ T cells have an immunodominance potential makes them an interesting target to stimulate by novel $CD8^+$ T cell-inducing influenza vaccines.

Following the Reviewer's comment, we have added the above statements to Discussion (line 419-428):

"*A potential low $CD8^+$ T cell frequency against this one NP145/HLA-A*68:01 epitope may not greatly affect the disease severity in individuals with additional HLAs capable of presenting universal influenza epitopes mounting robust influenza $CD8^+$ T cell responses against these universal epitopes. However, the frequency of the HLA-A*68:01 allomorph is especially high among the Indigenous populations globally including Southern America (http://www.allelefrequencies.net) and Australia (Clemens ICB 2016). These Indigenous populations often lack HLA allomorphs that present universal influenza epitopes and thus might lack influenza virus-specific $CD8^+$ T cell responses towards other HLAs (Quinones-parra 2014 PNAS). The fact that the $A68/NP_{145}$-specific $CD8^+$ T cells have an immunodominance potential makes*

them an interesting target to stimulate by novel $CD8^+$ T cell-inducing influenza vaccines."

Reviewers' Comments:

Reviewer #1:
Remarks to the Author:
The revised version of the paper addresses all of my previous comments and I recommend the paper be accepted for publication.

I do have two new minor comments listed below. Finally, I still think that generating Logo plots (even manually) from the tables in Figure 7 and 8 would be helpful for the readers, but I am not insisting on this for publication.

Two new minor comments:

1. Supp. Fig. 3 - I would urge more cautions statistical interpretation of this data (see: ASA statement on p-values. https://www.tandfonline.com/doi/pdf/10.1080/)00031305.2016.1154108?needAccess=true) actually I would argue that it is certainly possible that given additional subjects this trend will become significant.

2. While the cutoff for medium responders was modified to >12 the figures were not modified (3b, 4a and 5a).

Reviewer #2:
Remarks to the Author:
None

REVIEWERS' COMMENTS:

Reviewer #1 (Remarks to the Author):

The revised version of the paper addresses all of my previous comments and I recommend the paper be accepted for publication.

I do have two new minor comments listed below. Finally, I still think that generating Logo plots (even manually) from the tables in Figure 7 and 8 would be helpful for the readers, but I am not insisting on this for publication.

We thank the Reviewer for this comment. We acknowledge that Logo plots could strengthen the interpretation of the TCR data. Hence, we have previously published Logo plots in other papers including *Sant 2018 Frontiers in Immunology*. Unfortunately, the high variety of both expanded and non-expanded TCRs within and between donors does not allow us to include Logo plots for this manuscript.

Two new minor comments:

1. Supp. Fig. 3 - I would urge more cautions statistical interpretation of this data (see: ASA statement on p values https://www.tandfonline.com/doi/pdf/10.1080/)00031305.2016.1154108?needAccess=true) actually I would argue that it is certainly possible that given additional subjects this trend will become significant.

We agree with the Reviewer that our data might become statistically significant if we had the opportunity to include more donors for our study. Hence, we have now emphasized this In the Results section of our manuscript (Page 7, Line 219-222), which now reads:

*"Including additional donors could further strengthen the trend for an overall lower influenza virus-specific $CD8^+$ T cell response in HLA-A*68:01 positive individuals."*

2. While the cutoff for medium responders was modified to >12 the figures were not modified (3b, 4a and 5a).

We thank the Reviewer for this observation. We have now changed the figure headings in Figure 3b, 4a and 5a, which now read: "*Medium (>12) responders*" .

Reviewer #2 (Remarks to the Author):

None
