# Supplementary material for: Challenging immunodominance of influenza-specific CD8+ T cell responses restricted by the risk-associated HLA-A*68:01 allomorph
Source: Nat Commun. 2019 Dec 6;10:5579. doi: 10.1038/s41467-019-13346-4 (PMC6898063; doi:10.1038/s41467-019-13346-4)
Supplement: Supplementary file 3 — Peer Review File [file 41467_2019_13346_MOESM3_ESM.pdf]

nature research

Corresponding author(s): Katherine Kedzierska

Last updated by author(s): Nov 3, 2019

Reporting Summary

Nature Research wishes to improve the reproducibility of the work that we publish. This form provides structure for consistency and transparency in reporting. For further information on Nature Research policies, see Authors & Referees and the Editorial Policy Checklist.

Statistics

For all statistical analyses, confirm that the following items are present in the figure legend, table legend, main text, or Methods section.

n/a	Confirmed	
☐	☒	The exact sample size (*n*) for each experimental group/condition, given as a discrete number and unit of measurement
☐	☒	A statement on whether measurements were taken from distinct samples or whether the same sample was measured repeatedly
☐	☒	The statistical test(s) used AND whether they are one- or two-sided<br>*Only common tests should be described solely by name; describe more complex techniques in the Methods section.*
☐	☒	A description of all covariates tested
☐	☒	A description of any assumptions or corrections, such as tests of normality and adjustment for multiple comparisons
☐	☒	A full description of the statistical parameters including central tendency (e.g. means) or other basic estimates (e.g. regression coefficient) AND variation (e.g. standard deviation) or associated estimates of uncertainty (e.g. confidence intervals)
☐	☒	For null hypothesis testing, the test statistic (e.g. *F*, *t*, *r*) with confidence intervals, effect sizes, degrees of freedom and *P* value noted<br>*Give P values as exact values whenever suitable.*
☒	☐	For Bayesian analysis, information on the choice of priors and Markov chain Monte Carlo settings
☒	☐	For hierarchical and complex designs, identification of the appropriate level for tests and full reporting of outcomes
☒	☐	Estimates of effect sizes (e.g. Cohen's *d*, Pearson's *r*), indicating how they were calculated

Our web collection on statistics for biologists contains articles on many of the points above.

Software and code

Policy information about availability of computer code

Data collection	BD FACS DIVA, NCBI influenza resource database
Data analysis	FlowJo, Prism, SPSS statistics 25,TCRdist, Circos software, BioEdit, Ugene1.16.1, CCP4, XDS, MacPyMOL v1.7.6.3

For manuscripts utilizing custom algorithms or software that are central to the research but not yet described in published literature, software must be made available to editors/reviewers. We strongly encourage code deposition in a community repository (e.g. GitHub). See the Nature Research guidelines for submitting code & software for further information.

Data

Policy information about availability of data

All manuscripts must include a data availability statement. This statement should provide the following information, where applicable:

- Accession codes, unique identifiers, or web links for publicly available datasets
- A list of figures that have associated raw data
- A description of any restrictions on data availability

PDB accession number for the HLA-A*68:01-NP145 complex structure is 6PBH. TCR sequence data have been deposited into VDJdb [https://vdjdb.cdr3.net]. The source data underlying Figs 2-8, Supplementary Figs 2-4, Table 2 and Supplementary Table 3 are provided as a Source Data file. All other data are available from the authors upon request.

Field-specific reporting

Please select the one below that is the best fit for your research. If you are not sure, read the appropriate sections before making your selection.

☒ Life sciences ☐ Behavioural & social sciences ☐ Ecological, evolutionary & environmental sciences

For a reference copy of the document with all sections, see nature.com/documents/nr-reporting-summary-flat.pdf

Life sciences study design

All studies must disclose on these points even when the disclosure is negative.

Sample size	The sample size was determined by the availability of samples
Data exclusions	No data were excluded with the following exception: Donors who had a total number of less then 10 counted A68/CP145+CD8+ T cells within the whole enriched fraction were excluded for further phenotypic analysis as cell numbers were to low. This was indicated in the manuscript, see supplemental figure 2.
Replication	Experiments could not be replicated due to limited PBMC numbers, with the exception of donor 6 for A68/NP145 frequencies and phenotypes
Randomization	N/A
Blinding	Experiments were not blinded.

Reporting for specific materials, systems and methods

We require information from authors about some types of materials, experimental systems and methods used in many studies. Here, indicate whether each material, system or method listed is relevant to your study. If you are not sure if a list item applies to your research, read the appropriate section before selecting a response.

Materials & experimental systems

n/a	Involved in the study	
☐	☒	Antibodies
☒	☐	Eukaryotic cell lines
☒	☐	Palaeontology
☒	☐	Animals and other organisms
☐	☒	Human research participants
☒	☐	Clinical data

Methods

n/a	Involved in the study	
☒	☐	ChIP-seq
☐	☒	Flow cytometry
☒	☐	MRI-based neuroimaging

Antibodies

Antibodies used	We used commercially-available antibodies as per Material and Methods
Validation	Antibodies were validated by manufacturers and titrated in our laboratory prior to their use

Human research participants

Policy information about studies involving human research participants

Population characteristics	Please refer to Table 1 in the manuscript for details.
Recruitment	Samples were recruited through University of Melbourne (UoM), Australian Red Cross Blood Service (ARCBS), Deepdene Medical Clinic (DMC) (through co-author J. Crowe), Menzies School of Health Research (through co-authors A Miller and S Tong), Royal Melbourne Hospital (RMH) and Alfred Hospital (AH) (through co-authors A Cheng, T Kotsimbos and M Richards). Signed informed consents were obtained from all blood donors prior to study.
Ethics oversight	Human experiment work was approved by Human Research Ethics Committee (HREC) of the University of Melbourne (UoM) (Ethics ID #1443389.4) Nothern Territory Department of Health and Menzies School of Health Research (ID #HREC-2012-1928) For LIFT donors and HREC of Monash Health and Melbourne Health (ID #HRE/15/MonH/64) for RMG donors and Alfred Hospital (ID #280/14). All donors provided informed written consent.

Note that full information on the approval of the study protocol must also be provided in the manuscript.

Flow Cytometry

Plots

Confirm that:

☒ The axis labels state the marker and fluorochrome used (e.g. CD4-FITC).

☐ The axis scales are clearly visible. Include numbers along axes only for bottom left plot of group (a 'group' is an analysis of identical markers).

☐ All plots are contour plots with outliers or pseudocolor plots.

☒ A numerical value for number of cells or percentage (with statistics) is provided.

Methodology

Sample preparation	Samples were prepared as described in Methods
Instrument	BD LSR Fortessa was used for acquisition of data and BD FACS AriaIII for cell sorting
Software	BD FACS Diva, FlowJo
Cell population abundance	Only single cell sorting was performed, which was confirmed by the presence of single TCR strains
Gating strategy	Gating strategy has been described in supplementary figure 1

☒ Tick this box to confirm that a figure exemplifying the gating strategy is provided in the Supplementary Information.
